# Supplementary material for: A micro-architectured material as a pressure vessel for green mobility
Source: Nat Commun. 2024 Jan 8;15:353. doi: 10.1038/s41467-024-44695-4 (PMC10774278; doi:10.1038/s41467-024-44695-4)
Supplement: Supplementary file 1 — Supplementary Information [file 41467_2024_44695_MOESM1_ESM.pdf]

# SUPPLEMENTARY INFORMATION FOR

## A Micro-Architectured Material as a Pressure Vessel for Green Mobility

Yoon Chang Jeong[1], Seung Chul Han[1], Cheng Han Wu[1], Kiju Kang[1]*

[1]School of Mechanical Engineering, Chonnam National University; Gwangju, 61186, Republic of Korea

*Corresponding author. Email: kjkang@chonnam.ac.kr

**Supplementary Note 1:**

**Internal volumes per total weight of previous shellular specimens**

The following figures depict the three-dimensional geometry of the Ni-P shellular specimens, which were measured using a micro-CT[1]. This geometry was similar to those of the specimens used in the internal pressure experiments[2] except of the sealing caps, which were inevitably needed to confine compressed fluid in the interior space. Thus, Kolesnikova et al.[2] also used this geometry to build their FEA models for stress analysis.

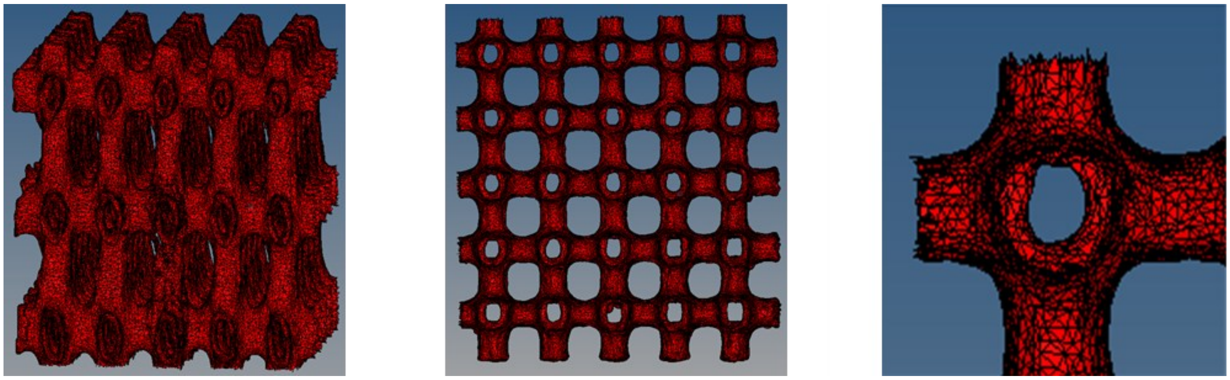

From these digital images, the surface area and internal volume of the specimens were calculated. By using these calculated values along with the shell thickness data provided in their paper, the internal volumes per total weight of each specimen were determined, as listed in Table S1.

**Table S1. Internal volumes per total weights of shellular and cylindrical pressure vessels**

Internal volumes per total weight and critical pressure of the Cu shellular specimens tested by Kolesnikova et al.[2] and theoretical values of the corresponding cylindrical pressure vessel.

| Shell thickness, *t* (mm) | Relative thickness, *t/D* | *Surface area, *A* ($mm^2$) | *Internal volume, $V_{in}$ ($mm^3$) | **Solid volume, $V_s$ ($mm^3$) | **Weight, m (g) | Internal volume per total weight ($mm^3$/g) | ***Internal volume per total weight ($mm^3$/g) | Critical pressure, $P_o$ (MPa) | ***Critical pressure, $P_o$ (MPa) |
|---|---|---|---|---|---|---|---|---|---|
| 0.005 | 0.0025 | 211.104 | 45.648 | 1.056 | 0.009415 | 4848 | 9300 | 0.1 | 0.525 |
| | | | | | | | | 0.29 | |
| 0.007 | 0.0035 | | | 1.478 | 0.01318 | 3463 | 6643 | 0.31 | 0.735 |
| | | | | | | | | 0.39 | |
| 0.01 | 0.005 | | | 2.111 | 0.01883 | 2424 | 4650 | 0.65 | 1.05 |
| | | | | | | | | 0.7 | |
| | | | | | | | | 0.77 | |
| 0.012 | 0.006 | | | 2.533 | 0.02260 | 2020 | 3875 | 0.79 | 1.26 |
| | | | | | | | | 0.87 | |
| | | | | | | | | 0.94 | |

* calculated from the digital topology measured using a micro-CT

** calculated from the surface area and the thickness data

*** theoretically estimated for the corresponding cylindrical pressure vessel as follows:

The internal volumes per total weight is given as follows:

$$\frac{\left(\frac{\pi}{4}D^2 l+\frac{\pi}{6}D^3\right)}{\rho t\left(\pi D l+\pi D^2\right)}=\frac{1}{12}\frac{D\left(3l+2D\right)}{\rho t\left(l+D\right)}=\frac{1}{12\rho}\left(\frac{t}{D}\right)^{-1}\left[3-\frac{D}{l+D}\right] .$$

Here, $\rho$ denotes the density of the constituent material, and the conventional cylindrical pressure vessel is assumed to have a straight section of length $l$ in the middle and hemispherical sealing caps on both sides. The yield pressure of a cylindrical pressure vessel is expressed as follows:

$$P_{\text{o}}=2\sigma_{\text{o}}\times\frac{t}{D}.$$

**Supplementary Note 2:**

**Finite Element Analyses (FEA)**

FEA modeling and procedures of shellulars with TPMSs

A TPMS sections space into two sub-volumes that are equivalent to, independent of, and intertwined with each other. A volume fraction is defined as the ratio of volumes of one sub-volume to the overall. In our design of the TPMS shellular pressure vessels, we used a constant volume fraction of $f = 0.5$, which means that the two sub-volumes take an identical volume in a unit cell. Finite element models of shellulars with the three TPMSs were prepared, based on the Surface Evolver software developed by Brakke[3]. The models were re-meshed with quadratic shell elements (S4) for FEA by using HyperMesh® (Altair Engineering, Inc., MI, USA). For the FEA, the cell size ($D$) and the mesh element size were fixed at 5 mm and approximately 70 μm, respectively, while the shell thickness varied in the range from $t = 0.5$ μm to 50 μm, (i.e., the relative thickness ranged from $t/D = 0.0001$ to 0.01). The Young's modulus, Poisson's ratio, and yield strength of the constituent material were given to be $E = 100$ GPa, $\nu = 0.3$, and $\sigma_o = 120$ MPa, respectively. Perfectly plastic behavior with no hardening was assumed after the initial yielding. To precisely determine yield pressure, the applied pressure was increased in steps of 1% of the expected yield pressure. Please refer to Wu[4] for the technical details.

Optimization of mesh size and element type

To secure the best accuracy with the minimal FEA run time, we investigated the effects of mesh size by using a double-chambered shellular vessel model composed of 3 × 3 × 3 cells. Figs. S1a–d depict the Mises stress distributions of the four models with mesh sizes of 0.5, 0.1, 0.07, and 0.02 mm, respectively. Fig. S1e summarizes the variation in their relative yield pressures as a function of mesh size. According to figure, the mesh size of 0.07 mm (70 μm) provided the best accuracy with the minimal run time. Hence, we used this mesh size for most models.

Four-node shell elements (S4 of Abaqus®), which are known to provide robust and accurate solutions under all loading conditions in thick and thin shell problems, were employed in the entire *t/D* range of 0.01–0.0001[5,6]. Their accuracy was validated by comparison with the analytical solutions of the conventional cylindrical and spherical pressure vessel models[3].

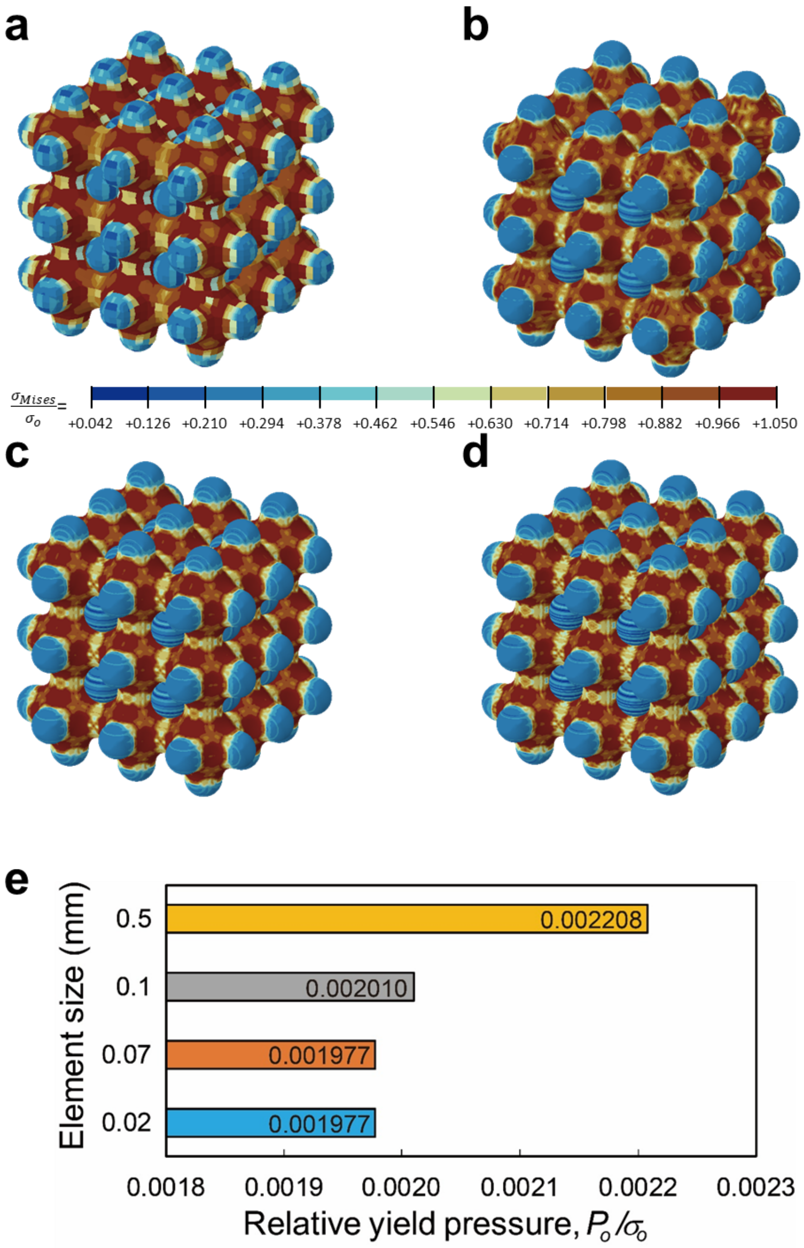


**Fig. S1. Effects of mesh size on FEA results.** Von Mises stress distribution in the four models of double-chambered shellular vessel with the mesh size of **a**, 0.5, **b**, 0.1, **c**,0.07, and **d**, 0.02 mm. **e**, Comparison of relative yield pressures in the four models of cold-stretched double-chambered shellular vessel with an identical shell thickness of $t = 0.001D$.

Full model versus unit cell model under periodic boundary conditions

Figures. S2a, b, and c show the von Mises stresses distributed on the full model of *P*-shellular with 3×3×3 cells, a single cell located at the center of the full model, and the unit cell under the symmetric periodic boundary conditions, respectively, under an identical internal pressure of $P = 0.000706\ \sigma_o$. The shell thicknesses of all the models were $t = 0.001\ D$. The stress distributions in Figs. S2b and c agree fairly well with each other. This agreement in the stress distributions between the single cell located at the center of the full model and the unit cell under the symmetric periodic boundary conditions was observed in *D*- and *F-RD*-shellulars, too. Hence, the unit cell models were used in subsequent investigations of the effect of cold stretching.

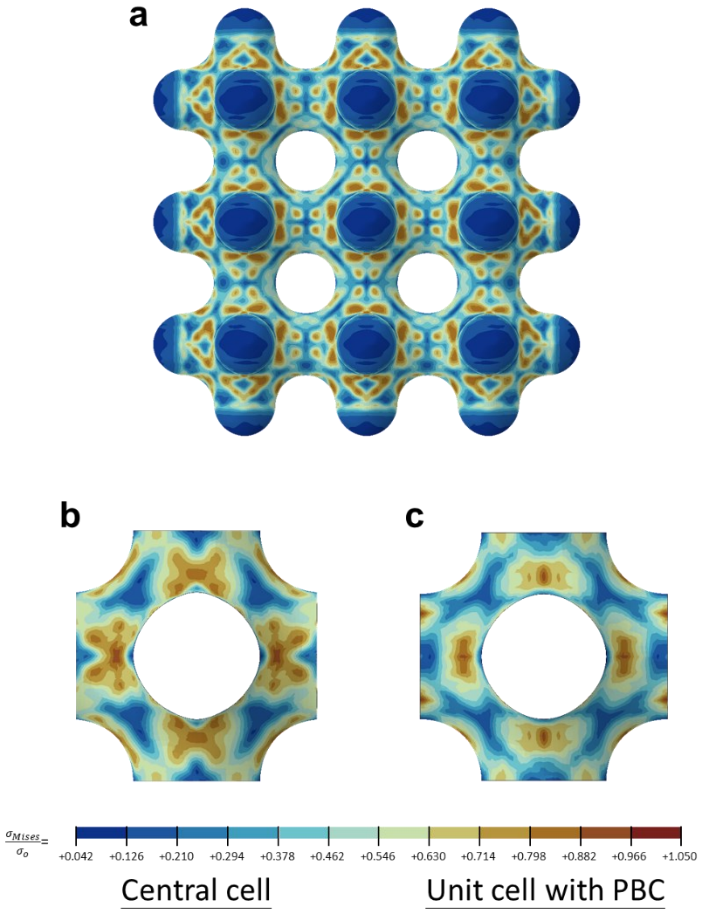


**Fig. S2. Full model versus unit cell model under periodic boundary conditions.** Von Mises stress distributed on **a**, a full model, **b**, a central cell of the full model, and **c**, a unit cell under symmetric periodic boundary conditions. All models are subjected to an identical internal pressure of $P = 0.000706\sigma_o$. Shell thicknesses are constant at $t = 0.001D$.

Effect of cold stretching

To establish the cold-stretched models, first, an excessive internal pressure was applied to each unit cell model under the periodic boundary conditions beyond a yield pressure, at which the von-Mises stress anywhere in the model first reaches the yield strength of the constituent material, until it plastically yields in the surface area over 85 %. Then, the pressure was released, and the residual stress was removed from the deformed configuration. Figure S3 shows the von Mises stress distribution at the yield pressure on the original models (left) and cold stretched models (right) with $t/D = 0.001$ for the three shellulars. Although unclear in the figure, the plastic deformation due to cold stretching mainly occurred around the midpoints between the openings of each model. The values of yield pressures before and after cold stretching are marked below for the three models. The cold-stretched models revealed more uniform stress distributions than the original models, and their yield pressures were substantially higher than those of the original models. In Fig. S4a, the relative yield pressures, $P_o/\sigma_o$, estimated for the three models are plotted against the relative thickness, $t/D$, which were fitted as follows:

$$\frac{P_o}{\sigma_o} = 1.357 \times \left(\frac{t}{D}\right)^{0.9358} \quad \text{for } P\text{-shellular,} \qquad \text{--- (1)}$$

$$\frac{P_o}{\sigma_o} = 3.0705 \times \left(\frac{t}{D}\right)^{1.224} \quad \text{for } D\text{-shellular, and}$$

$$\frac{P_o}{\sigma_o} = 1.1168 \times \left(\frac{t}{D}\right)^{0.8665} \quad \text{for } F\text{-}RD\text{-shellular.}$$

In Fig. S4b, the *EPV*s of the three shellulars are compared under two given yield pressures, $P_o = 0.01\ \sigma_o$ and $0.002\ \sigma_o$. The *P*-shellulars, particularly after cold-stretched, have the highest *EPV*s.

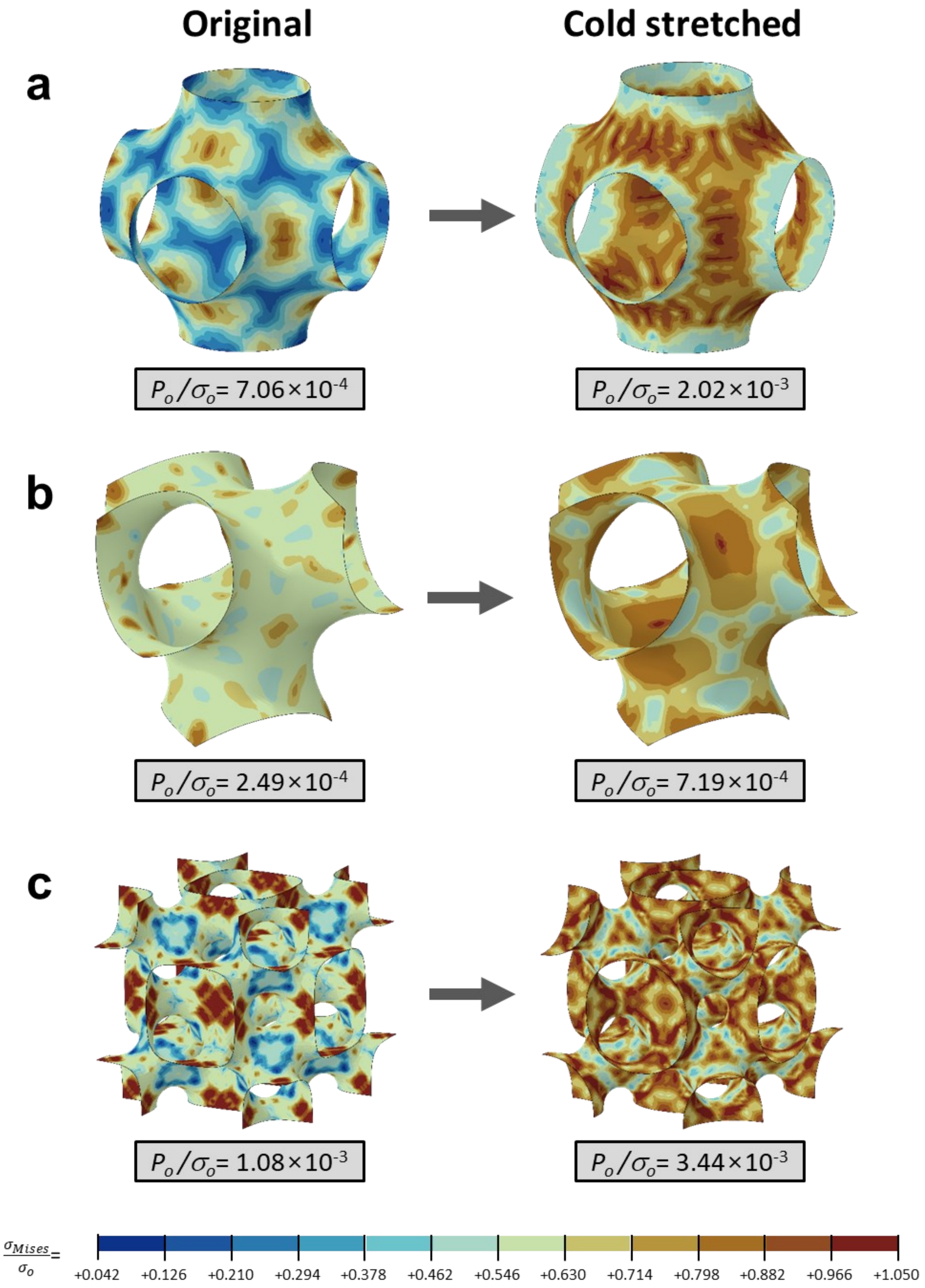


**Fig. S3. Effects of cold stretching estimated by FEA.** Von Mises stress distributions at ~~the~~ yield pressures on the original models (left) and cold-stretched models (right) with *t/D*=0.001 of **a**, *P*-, **b**, *D*-, and **c**, *F-RD*-shellulars.

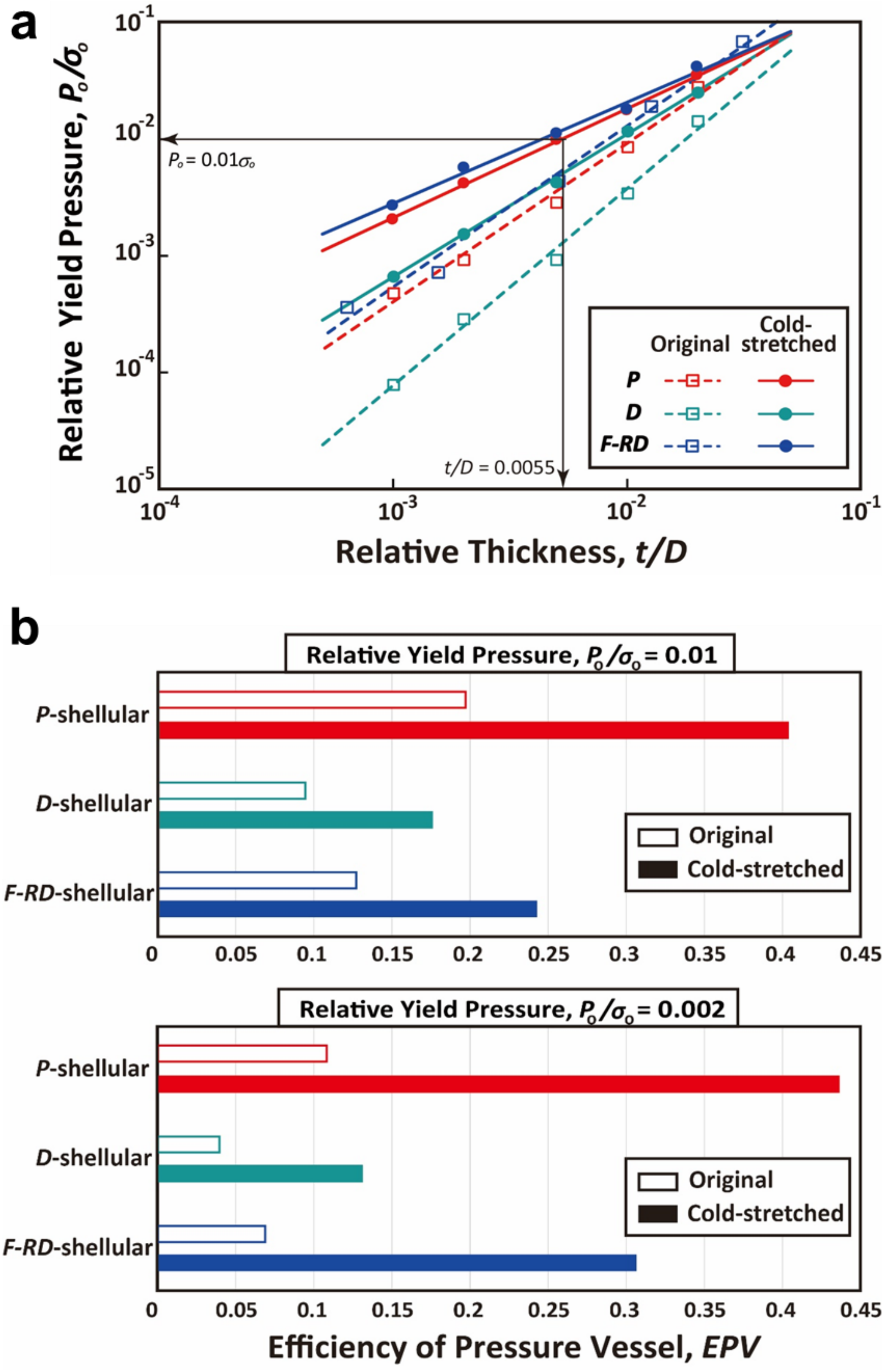


**Fig. S4. Relative yield pressures and *EPVs* for three types of TPMS shellulars.**

**a**, Relative yield pressures plotted against relative thickness ($t/D$) and **b**, comparison of *EPV*s under two given yield pressures of $P_o = 0.01\ \sigma_o$ and $0.002\ \sigma_o$. The yield pressures and *EPV*s were estimated from FEA for the original and cold-stretched unit cell models for *P*-, *D*-, and *F-RD* shellulars.

Effects of shell thickness and yield pressure on *EPV*

According to Eq. (1), the relative yield pressure, $P_o/\sigma_o$, of a *P*-shellular is almost linearly dependent on the relative thickness, $t/D$. And also, the internal volume, $V_{in}$, and the solid volume, $V_s = At$, are proportional to $D^3$ and $D^2$, respectively. Specifically,

$$V_{in} = 0.5D^3 \text{ and} \\ A = 2.34 \times D^2. \qquad \text{--- (S1)}$$

The formula for calculating $A$ is cited from Nguyen et al.[7]. Hence, simply by combining Eqs. (1), (2), and (S1), the *EPV* of a *P*-shellular composed of a sufficiently large number of cells is expressed as follows.

$$EPV = 0.29\left(\frac{t}{D}\right)^{-0.0642} = 0.2961\left(\frac{P_o}{\sigma_o}\right)^{-0.0686} \approx \text{constant}$$

Thus, the *EPV* of a *P*-shellular is almost constant regardless of the relative thickness or the relative yield pressure. To be specific, the *EPV* is 0.41 at $P_o/\sigma_o = 0.01$, varying from 0.48 to 0.35 as $t/D$ varies by 100 times in the range from 0.001 to 0.1, respectively. Please remember that the equations in this section were derived for the unit cell model of *P*-shellular and the effects of sealing caps were ignored.

Effect of cell size

The three models depicted in Fig. 2 in the main paper were designed to have hemispherical sealing caps on their outer openings. The cell size, $D$, of each model was determined depending on the number of cells such that its internal volume was the same as that of a spherical pressure vessel with a 1 m diameter. The shell thickness, $t$, was then calculated according to Eq. (1) for a constant yield pressure of $P_o = 0.01\ \sigma_o$. Specifically, the relative thicknesses were set to $t/D$ = 0.0055 = 4.9mm/909mm, 1.8mm/325mm, and 0.6mm/111mm for the models with the single, 3×3×3, and 9×9×9 cells, respectively. As shown in Figs. 2a, b, and c, the stress distributions were almost identical to each other as expected despite the differences in the cell size and shell

thickness. We applied the same approach to estimate the cell size and shell thickness of the model with 100×100×100 cells (i.e., one million cells) to resist the yield pressure of $P_o$ = 0.01 $\sigma_o$. Table S2 lists the dimensions and *EPV*s of the *P*-shellular vessels with the single, 3×3×3, 9×9×9, and 100×100×100 cells in comparison with those of the conventional cylindrical and spherical pressure vessels.

Single-chambered versus double-chambered vessels

The bottom in Table S2 (highlighted in yellow) lists the dimensions and *EPV* of the double-chambered counterparts with 3×3×3, 9×9×9, and 100×100×100 cells. Here, the overall size, cell size, shell thickness of each vessel, and yield pressure were set to be the same as those of the single-chambered vessels with the same number of cells. As the total number of cells increased, namely, as the unit cell size decreased, the number of cells in the second sub-volume converged to that in the first sub-volume, and a portion of the sealing caps in the total weight decreased. Consequently, the internal volume and *EPV* of the double-chambered vessel approached twice that of its single-chambered counterpart.

To check if the yield pressure of the double-chambered vessel is the same as that of its single-chambered counterpart and to examine the feasibility of the double-chambered shellular as a pressure vessel, we carried out additional FEA with $t/D$ = 0.001. The results are shown in Fig. S5, in which the left and right columns display the von Mises stress distributions on the inner surfaces of the first and second sub-volumes, respectively, as illustrated in the insets in the middle row. Specifically, Figs. S5a and b show the stress distributions when the first and the second sub-volumes were individually pressurized with $P$ = 0.002 $\sigma_o$, respectively. The two stress distributions were very similar to each other regardless of which sub-volume was pressurized. Figures. S5c and d also show the stress distribution when both sub-volumes were simultaneously pressurized. In the two figures, when the stresses in one sub-volume are shown, those in the other sub-volume are omitted and displayed in gray. The stress distribution on the outer shells of the first sub-volume was very similar to that observed when

only the first sub-volume was pressurized, shown in Fig. S5a. In contrast, the stress distribution on the inner shells sharing with the second sub-volume was dissimilar from that observed when only the first sub-volume was pressurized; thus, the stresses were hardly concentrated. The overall stress distribution in the second sub-volume was much lower than that observed when only the second sub-volume was pressurized, shown in Fig. S5b. This means that the second sub-volume can resist the higher pressure than the first sub-volume. However, if the two sub-volumes are connected through small holes penetrating the interfacial shell between them (like in the tested specimens in this study) and equally pressurized, the yield pressure of the double-chambered shellular becomes the same as that of its single-chambered counterpart.

**Table S2. *EPV*s of *P*-shellular pressure vessels.**
Dimensions and *EPV*s of conventional spherical and cylindrical pressure vessels and the *P*-shellular pressure vessels with four different cell sizes, designed to have a constant internal volume under a constant yield pressure of $P_o = 0.01\ \sigma_o$.

<table>
<tr><th colspan="2"></th><th>Overall size</th><th>Diameter or cell size, $D$ (m)</th><th>Shell thickness, $t$ (mm)</th><th>Surface area, $A$ ($m^2$)</th><th>Internal volume, $V_{in}$ ($m^3$)</th><th>Solid volume, $V_s$ ($10^{-3}\ m^3$)</th><th>*EPV*</th></tr>
<tr><td colspan="2">Spherical</td><td>1.000</td><td>1.000</td><td>2.5</td><td>3.142</td><td rowspan="6">0.5236</td><td>7.9</td><td>0.67</td></tr>
<tr><td colspan="2">Cylindrical</td><td>1.474</td><td>0.737</td><td>3.7</td><td>3.411</td><td>12.6</td><td>0.42</td></tr>
<tr><td>*P*-shellular single cell</td><td rowspan="4">Single chambered</td><td>1.364</td><td>0.909</td><td>5.0</td><td>3.884</td><td>21.6</td><td>0.27</td></tr>
<tr><td>*P*-shellular 3×3×3 cells</td><td>1.137</td><td>0.325</td><td>1.8</td><td>8.908</td><td>16.0</td><td>0.34</td></tr>
<tr><td>*P*-shellular 9×9×9 cells</td><td>1.057</td><td>0.111</td><td>0.6</td><td>23.47</td><td>14.1</td><td>0.37</td></tr>
<tr><td>*P*-shellular million cells</td><td>1.019</td><td>0.010</td><td>$5.4 \times 10^{-2}$</td><td>234.1</td><td>13.2</td><td>0.39</td></tr>
<tr><td>*P*-shellular 3×3×3 cells</td><td rowspan="3">Double chambered</td><td>1.137</td><td>0.325</td><td>1.8</td><td>9.903</td><td>0.6877</td><td>17.4</td><td>0.40</td></tr>
<tr><td>*P*-shellular 9×9×9 cells</td><td>1.057</td><td>0.111</td><td>0.6</td><td>25.34</td><td>0.8933</td><td>15.2</td><td>0.59</td></tr>
<tr><td>*P*-shellular million cells</td><td>1.019</td><td>0.010</td><td>$5.4 \times 10^{-2}$</td><td>245.5</td><td>1.0317</td><td>13.4</td><td>0.77</td></tr>
</table>

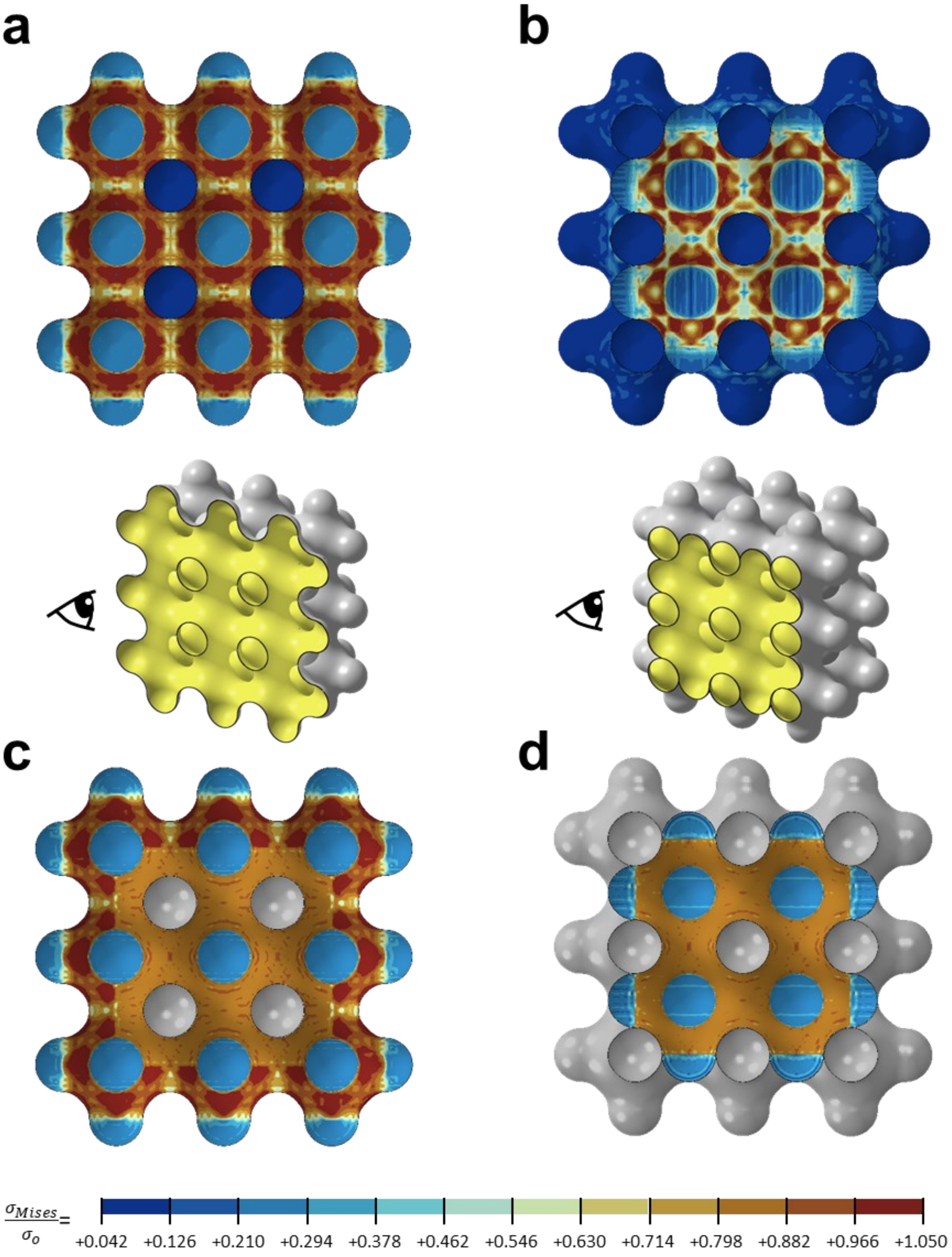


**Fig. S5. Stress distribution in double-chambered *P*-shellular vessels.**

Von Mises stresses distribution on a middle section of a double-chambered full model ($t/D$ = 0.001) of 3×3×3 cells with **a**, a first sub-volume only pressured, **b**, a second sub-volume only pressured, **c**, **d**, both sub-volumes pressured with $P = 0.002\ \sigma_o$.

**Supplementary Note 3:**

**General Solutions for Efficiency of Pressure Vessel (*EPV*)**

Conventional spherical and cylindrical pressure vessels

The yield pressures for spherical and cylindrical pressure vessels are given by

$$\frac{P_o}{\sigma_o} = 4 \times \frac{t}{D} \quad \text{--- (S2)}$$

and

$$\frac{P_o}{\sigma_o} = 2 \times \frac{t}{D}, \quad \text{--- (S3)}$$

respectively. Hence, the *EPV*, defined by Eq. (2), can be derived using Eqs. (S2) and (S3) for the conventional spherical and cylindrical pressure vessels, respectively, as follows.

$$EPV = \frac{P_o}{\sigma_o}\frac{\pi D^3}{6\pi D^2 t} = \frac{P_o}{\sigma_o}\frac{D}{6t} = \frac{2}{3} \quad \text{--- (S4)}$$

and

$$EPV = \frac{P_o}{\sigma_o}\frac{\left(\frac{\pi}{4}D^2 l + \frac{\pi}{6}D^3\right)}{t\left(\pi D l + \pi D^2\right)} = \frac{1}{12}\frac{P_o}{\sigma_o}\frac{D(3l+2D)}{t(l+D)} = \frac{1}{2} - \frac{D}{6(l+D)}. \quad \text{--- (S5)}$$

Here, the conventional cylindrical pressure vessel is assumed to have a straight section of length, *l*, in the middle and hemispherical sealing caps on both sides. According to Eq. (S5), the *EPV* varies slowly from 5/12 to 1/2 as the straight section length varies from $l = D$ to $l = \infty$. Thus, the *EPV*s for the conventional spherical and cylindrical pressure vessels are almost constant regardless of the size and material.

Single-chambered *P*-shellular pressure vessel

The internal volume and surface area of a single-chambered *P*-shellular pressure vessel with hemispherical sealing caps on its outer boundaries are expressed as follows.

$$\begin{aligned} V_{in} &= V_{shellular} + V_{caps} \\ &= 0.5N^3D^3 + \frac{\pi}{16}N^2D^3 = \left(0.5 + \frac{\pi}{16N}\right)N^3D^3 \end{aligned} \qquad \text{--- (S6)}$$

$$\begin{aligned} A &= A_{shellular} + A_{caps} \\ &= 2.34N^3 \times D^2 + \frac{3\pi}{4}N^2D^2 = \left(2.34N + \frac{3\pi}{4}\right)N^2D^2 \end{aligned} \qquad \text{--- (S7)}$$

Here, the subscripts, *shellular* and *caps*, denote the properties of the shellular in the *P*-surface and hemispherical caps. The shell thickness for $P_o/\sigma_o = 0.01$ can be estimated using Eq. (1) as follows.

$$\begin{aligned} \frac{P_o}{\sigma_o} &= 1.357 \times \left(\frac{t}{D}\right)^{0.9358} \\ t &= D\left(\frac{0.01}{1.357}\right)^{1/0.9358} = 0.00526D \end{aligned} \qquad \text{--- (S8)}$$

The solid volume is expressed as follows.

$$\begin{aligned} V_s &= At = \left(2.34N + \frac{3\pi}{4}\right)N^2D^2 \times 0.00526D \\ &= 0.00526 \times \left(2.34N + \frac{3\pi}{4}\right)N^2D^3 \end{aligned} \qquad \text{--- (S9)}$$

Hence, the efficiency of pressure vessel (*EPV*) for $P_o/\sigma_o = 0.01$ is

$$EPV = \frac{P_oV_{in}}{\sigma_oV_s} = \frac{0.01 \times \left(0.5 + \frac{\pi}{16N}\right)}{0.00526 \times \left(2.34 + \frac{3\pi}{4N}\right)} = \frac{\left(0.5 + \frac{\pi}{16N}\right)}{0.526 \times \left(2.34 + \frac{3\pi}{4N}\right)}$$

$$EPV = 0.475 \times \frac{(8N + \pi)}{(9.36N + 3\pi)} \qquad \text{--- (3)}$$

For example, for $N = 1, 3, 9$, and 100, the $EPV = 0.28, 0.34, 0.38$, and 0.40, respectively. And for $N =$ infinitive, $EPV = 0.475 \times 8 \div 9.36 = 0.406$. Also, note that Eq. (1) reveals that $P_o/\sigma_o$ is almost linear to $t/D$. Consequently,

$$EPV = \frac{P_o V_{in}}{\sigma_o V_s} = 1.357\left(\frac{t}{D}\right)^{0.9358} \times \frac{0.5N^3D^3}{2.34N^3D^2t} = 0.29\left(\frac{t}{D}\right)^{-0.0642} \approx \text{constant} . \qquad \text{--- (S10)}$$

Double-chambered $P$-shellular pressure vessel

The internal volume and surface area of a double-chambered $P$-shellular pressure vessel with hemispherical sealing caps on its outer boundaries are expressed as follows.

$$\begin{aligned} V_{in} &= V_{shellular1} + V_{shellular2} + V_{caps1} + V_{caps2} \\ &= 0.5N^3D^3 + 0.5(N-1)^3 D^3 + \frac{\pi}{16}N^2D^3 + \frac{\pi}{16}(N-1)^2 D^3 \\ &= \left[\frac{1}{2}\left(1+\left(\frac{N-1}{N}\right)^3\right) + \frac{\pi}{16N}\left(1+\left(\frac{N-1}{N}\right)^2\right)\right] \times N^3D^3 \end{aligned} \qquad \text{--- (S11)}$$

$$\begin{aligned} A &= A_{shellular1} + A_{caps1} + A_{caps2} \\ &= 2.34N^3 \times D^2 + \frac{3\pi}{4}N^2D^2 + \frac{3\pi}{4}\left(\frac{N-1}{N}\right)^2 N^2D^2 \\ &= \left[2.34N + \frac{3\pi}{4}\left(1+\left(\frac{N-1}{N}\right)^2\right)\right] N^2D^2 \end{aligned} \qquad \text{--- (S12)}$$

Subscripts 1 and 2 denote the first and second sub-volumes, respectively. The shell thickness for $P_o/\sigma_o = 0.01$ can be estimated using Eq. (1) as follows.

$$\frac{P_o}{\sigma_o} = 1.357 \times \left(\frac{t}{D}\right)^{0.9358}$$

$$t = D\left(\frac{0.01}{1.357}\right)^{1/0.9358} = 0.00526D$$

The solid volume is expressed as follows.

$$V_s = At = \left(2.34N + \frac{3\pi}{4}\left(1+\left(\frac{N-1}{N}\right)^2\right)\right)N^2D^2 \times 0.00526D$$
$$= 0.00526\times\left(2.34N + \frac{3\pi}{4}\left(1+\left(\frac{N-1}{N}\right)^2\right)\right)N^2D^3 \qquad \text{--- (S13)}$$

Thus, the *EPV* for $P_o/\sigma_o = 0.01$ is given as follows.

$$EPV = \frac{P_oV_{in}}{\sigma_oV_s} = \frac{0.01\times\left[\frac{1}{2}\left(1+\left(\frac{N-1}{N}\right)^3\right)+\frac{\pi}{16N}\left(1+\left(\frac{N-1}{N}\right)^2\right)\right]}{0.00526\times\frac{1}{N}\left(2.34N+\frac{3\pi}{4}\left(1+\left(\frac{N-1}{N}\right)^2\right)\right)}$$

$$EPV = 0.475\times\frac{8N\left(1+\left(\frac{N-1}{N}\right)^3\right)+\pi\left(1+\left(\frac{N-1}{N}\right)^2\right)}{9.36N+3\pi\left(1+\left(\frac{N-1}{N}\right)^2\right)} \qquad \text{--- (4)}$$

For example, for $N = 3$, 9, and 100, the *EPV* = 0.41, 0.60, and 0.79, respectively. And for $N$ = infinitive, *EPV* = 0.475×16÷9.36 = 0.812.

**Supplementary Note 4:**

**General Solutions for Solid Volumes**

Single conventional spherical vessel

The internal volume and area are expressed as follows:

$$\begin{aligned} V_o &= \frac{\pi}{6} D_o^{\,3} \\ A_o &= \pi D_o^{\,2} \end{aligned} \qquad \text{--- (S14)}$$

By substituting Eq. (S2), the solid volume of a single spherical pressure vessel can be given as

$$V_{so} = A_o t_o = \pi D_o^{\,2} \left( \frac{P_o}{\sigma_o} \right) \frac{D_o}{4} = \frac{\pi}{4} \left( \frac{P_o}{\sigma_o} \right) D_o^{\,3} . \qquad \text{--- (S15)}$$

Hence, for $P_o/\sigma_o = 0.01$, the solid volume is given as

$$V_{so} = \frac{\pi}{4} (0.01) D_o^{\,3} = 0.00785\, D_o^{\,3} .$$

Matrix of spherical vessels with $N \times N \times N$ cells

If their diameter is

$$D_1 = \frac{D_o}{N} ,$$

the internal volume and area of the matrix are, respectively,

$$\begin{aligned} V_1 &= \frac{\pi}{6} \left( \frac{D_o}{N} \right)^3 \times N^3 = \frac{\pi}{6} D_o^{\,3} = V_o \\ A_1 &= \pi \left( \frac{D_o}{N} \right)^2 \times N^3 = \pi N D_o^{\,2} = N A_o \end{aligned} . \qquad \text{--- (S16)}$$

By substitution of Eq. (S2), the solid volume of the matrix of spherical pressure vessels can be expressed as

$$V_{s1} = A_1 t_1 = \pi N D_o^{\ 2}\left(\frac{P_o}{\sigma_o}\right)\frac{D_1}{4} = \pi N D_o^{\ 2}\left(\frac{P_o}{\sigma_o}\right)\frac{D_o}{4N} = \frac{\pi}{4}\left(\frac{P_o}{\sigma_o}\right)D_o^{\ 3} = V_{so} \qquad \text{--- (S17)}$$

That is, the solid volume of the matrix is constant, regardless of the number of cells in one direction, *N*.

<u>Single shellular vessel</u>

From Eq. (S6), the internal volume of a single shellular vessel is expressed by

$$V_{in} = \left(0.5 + \frac{\pi}{16}\right)D^3 \qquad \text{--- (S18)}$$

If the internal volume of a single shellular vessel is set to equal to that of a single spherical vessel, the cell size of the single shellular vessel is related to the diameter of the sphere, as follows:

$$V_{in} = \left(0.5 + \frac{\pi}{16}\right)D^3 = \frac{\pi}{6}D_o^{\ 3} = V_o$$

$$D = \left(\frac{\frac{\pi}{6}}{0.5 + \frac{\pi}{16}}\right)^{\frac{1}{3}} D_o = 0.9093 D_o \qquad \text{--- (S19)}$$

From Eq. (S7), the area of the single shellular vessel is expressed as follows:

$$\begin{aligned} A &= A_{shellular} + A_{caps} \\ &= 2.34 \times D^2 + \frac{3\pi}{4}D^2 = \left(2.34 + \frac{3\pi}{4}\right)D^2 = \left(2.34 + \frac{3\pi}{4}\right)\times 0.9093^2 D_o^{\ 2} \qquad \text{--- (S20)} \\ &= 3.8829 D_o^{\ 2} \end{aligned}$$

Therefore, by substituting Eq. (S8), the solid volume of the single shellular vessel needed for $P_o/\sigma_o = 0.01$ is expressed as

$$V_s = At = \left(2.34 + \frac{3\pi}{4}\right) D^2 \times 0.00526D = 0.02470D^3 = 0.01857{D_o}^3 . \qquad \text{--- (S21)}$$

Double-chambered shellular vessel with $N \times N \times N$ cells

The internal volume of a double-chambered shellular vessel is expressed as Eq. (S11)

$$\begin{aligned} V_{in} &= V_{shellular1} + V_{shellular2} + V_{caps1} + V_{caps2} \\ &= 0.5N^3D^3 + 0.5(N-1)^3 D^3 + \frac{\pi}{16} N^2 D^3 + \frac{\pi}{16}(N-1)^2 D^3 \qquad \text{--- (S11)} \\ &= \left[\frac{1}{2}\left(1 + \left(\frac{N-1}{N}\right)^3\right) + \frac{\pi}{16N}\left(1 + \left(\frac{N-1}{N}\right)^2\right)\right] \times N^3 D^3 \end{aligned}$$

If the internal volume of the double-chambered shellular vessel is set to be equal to that of a single spherical vessel, the cell size of the single shellular vessel is related to the diameter of the sphere, as follows:

$$V_{in} = \left[\frac{1}{2}\left(1 + \left(\frac{N-1}{N}\right)^3\right) + \frac{\pi}{16N}\left(1 + \left(\frac{N-1}{N}\right)^2\right)\right] \times N^3 D^3 = \frac{\pi}{6}{D_o}^3 = V_o$$

$$D = \left[\frac{\frac{\pi}{6}}{\frac{1}{2}\left(1 + \left(\frac{N-1}{N}\right)^3\right) + \frac{\pi}{16N}\left(1 + \left(\frac{N-1}{N}\right)^2\right)}\right]^{\frac{1}{3}} \frac{1}{N} D_o \qquad \text{--- (S22)}$$

From Eq. (S12), the area of the double-chambered shellular vessel is expressed as follows:

$$A = \left[2.34N + \frac{3\pi}{4}\left(1 + \left(\frac{N-1}{N}\right)^2\right)\right] N^2 D^2$$

Therefore, by substituting Eq. (S8), the solid volume of the double-chambered shellular vessel needed for $P_o/\sigma_o = 0.01$ is expressed as

$$V_s = At = \left[2.34N + \frac{3\pi}{4}\left(1+\left(\frac{N-1}{N}\right)^2\right)\right]N^2D^2 \times 0.00526D$$
$$= 0.00526\left[2.34N + \frac{3\pi}{4}\left(1+\left(\frac{N-1}{N}\right)^2\right)\right]N^2D^3 \qquad \text{--- (S23)}$$

**Supplementary Note 5:**

**Validation of Eqs. (S6), (S7), (S11), and (S12)**

These equations describe the internal volume and surface area of the single and double-chambered shellular vessels, and they are used to calculate *EPV* and $V_s$. Thus, their accuracy is crucial. We validate these equations by comparing the internal volume and surface area values calculated using them to those of the corresponding computer-aided design (CAD) models with 3 × 3 × 3 and 9 × 9 × 9 cells. Table S3 compares the results. The internal volume and surface area estimated using these equations agree well with those calculated from the CAD models. The errors are smaller than 0.3%, demonstrating the accuracy of the equations.

**Table S3. Validation of Eqs. (S6), (S7), (S11), and (S12)**
Internal volumes and surface areas of single- and double-chambered shellular vessel models with 3 × 3 × 3 and 9 × 9 × 9 cells estimated using Eqs. (S6), (S7), (S11), and (S12); those calculated from the corresponding CAD models; and the errors between these two sets of values.

| | Number of cells in a row, *N* | Cell size, *D* (m) | | Internal volume, $V_{in}$ ($m^3$) | Surface area, *A* ($m^2$) | *Error in $V_{in}$ (%) | *Error in *A* (%) |
|---|---|---|---|---|---|---|---|
| Single chambered shellular vessels | 3 | 0.2967 | Equation | 0.3986 | 7.427 | -0.2011 | -0.1213 |
| | | | CAD model | 0.3978 | 7.418 | | |
| | 9 | 0.09310 | Equation | 0.5235 | 8.257 | -0.2298 | -0.2550 |
| | | | CAD model | 0.5223 | 8.236 | | |
| Double chambered shellular vessels | 3 | 0.2967 | Equation | 0.3069 | 16.44 | -0.0652 | 0.1215 |
| | | | CAD model | 0.3067 | 16.46 | | |
| | 9 | 0.09310 | Equation | 0.5235 | 17.75 | -0.0956 | -0.05637 |
| | | | CAD model | 0.5230 | 17.74 | | |

* calculated by $\frac{(Equation)-(CAD\ model)}{(CAD\ model)} \times 100$

**Supplementary Note 6:**

**Specimen Preparation**

The expected high performance of a *P*-shellular pressure vessel is owing to its unique geometry with constant mean curvature. Thus, the specimens should be fabricated with high precision. Another technical challenge is to achieve a uniform shell thickness without any defects over the entire body of each specimen. Fortunately, the surface of a TPMS is continuous and free of self-intersections, thus providing an important advantage. Hence, a shellular specimen could be fabricated by a single deposition of a metallic layer on a polymer template by electroless plating, followed by etching out the template. Copper (Cu) was selected as the constituent material of the metallic layer to provide sufficient ductility, which is essential for cold stretching, and it was deposited on the template through electroless plating. The *P*-shellular specimens were prepared with a cell size of 5 mm, shell thickness of 4 to 110 μm, and 3×3×3 cells. More detailed fabrication processes are as follows.

First, a negative template of a water-soluble polymer, polyvinyl alcohol (PVA), was prepared using a low-cost 3D printer (Ultimaker S3, Ultimaker B.V., Netherland), which uses fused deposition modeling with 0.2 mm layer thickness. Secondly, two components (powder and liquid) of a cold-curing resin (CCR, Technovit® 5071 and universal liquid, Heraeus Kulzer GmbH, Germany) were mixed in a weight ratio of 4:3 and poured onto the negative PVA template placed within a silicon rubber container. After 20 min under the pressure of 0.6MPa applied to prevent void formation, the mixture was polymerized to poly-methyl methacrylate (PMMA); subsequently, the outer side faces were polished to expose the interior PVA template. The PVA template was then etched out in the water for 24 h at 60 °C. The remaining positive PMMA template was washed with deionized (DI) water and dried in the air before the sprues were trimmed. Figure S6a shows the negative template of PVA as printed. Figures S6b and c show the template with the outer side faces polished to expose the interior PMMA and the positive PMMA template obtained after PVA was etched out, respectively.

After that, the flat-topped bumps on the side faces were ground into dome-shaped caps, and the surface of the PMMA template was then smoothened through a two-stage chemical process, called Han's treatment[8]. Namely, the template was dipped in a mixture of methylene chloride and ethanol at 3:2 (good solvent) for 3 min, and then dipped in a mixture of tetrahydrofuran (THF) and xylene at 4:1 (poor solvent) for 1 min. Han's treatment was repeated three times. Finally, before Cu plating, the PMMA template was dried in the air for 40 min to allow the poor solvent on the surfaces to volatilize and then dipped in a non-solvent, methanol, for 7 min to harden by completely removing the solvents remaining in the template and to provide roughness on the surfaces.

The electroless plating of Cu was conducted in four steps that consisted of pre-dip, activator coating, tin removal, and Cu plating. Specifically, first, the template was dipped in an aqueous solution of HCl for 3 min, and then dipped in another aqueous solution of PT-Activator (Young-In Plachem Co. Ltd., Korea) and HCl for 5 min at 40 °C for the activator coating. Thereafter, the template was dipped in 10% NaOH aqueous solution (accelerator) for 5 min to remove tin on the surface. Cu plating on the surface was finally performed according to the commercial process based on ELC-250 solution (Young-In Plachem Co. Ltd., Korea) at 70°C and 11.8 ~ 12.1 pH levels. The plating rate was approximately 4 μm/h. Thus, the Cu layer thicknesses of $t$ = 5, 16.25, 27.5, 38.75, and 50 μm were obtained by controlling the plating time as 1h 15min, 4h 5min, 6h 55min, 9h 45min, and 12h 30min, respectively. Figures S6d, e, and f show the positive templates after the three steps, i.e., forming the caps by grinding the flat-topped bumps on the side faces, Han's treatment, and Cu plating, respectively, and the magnified images of the red squares are shown in Figs. S6g, h, and i, respectively. Figure S6j shows the schematic of the entire process conducted to obtain a shellular specimen for the internal pressure test.

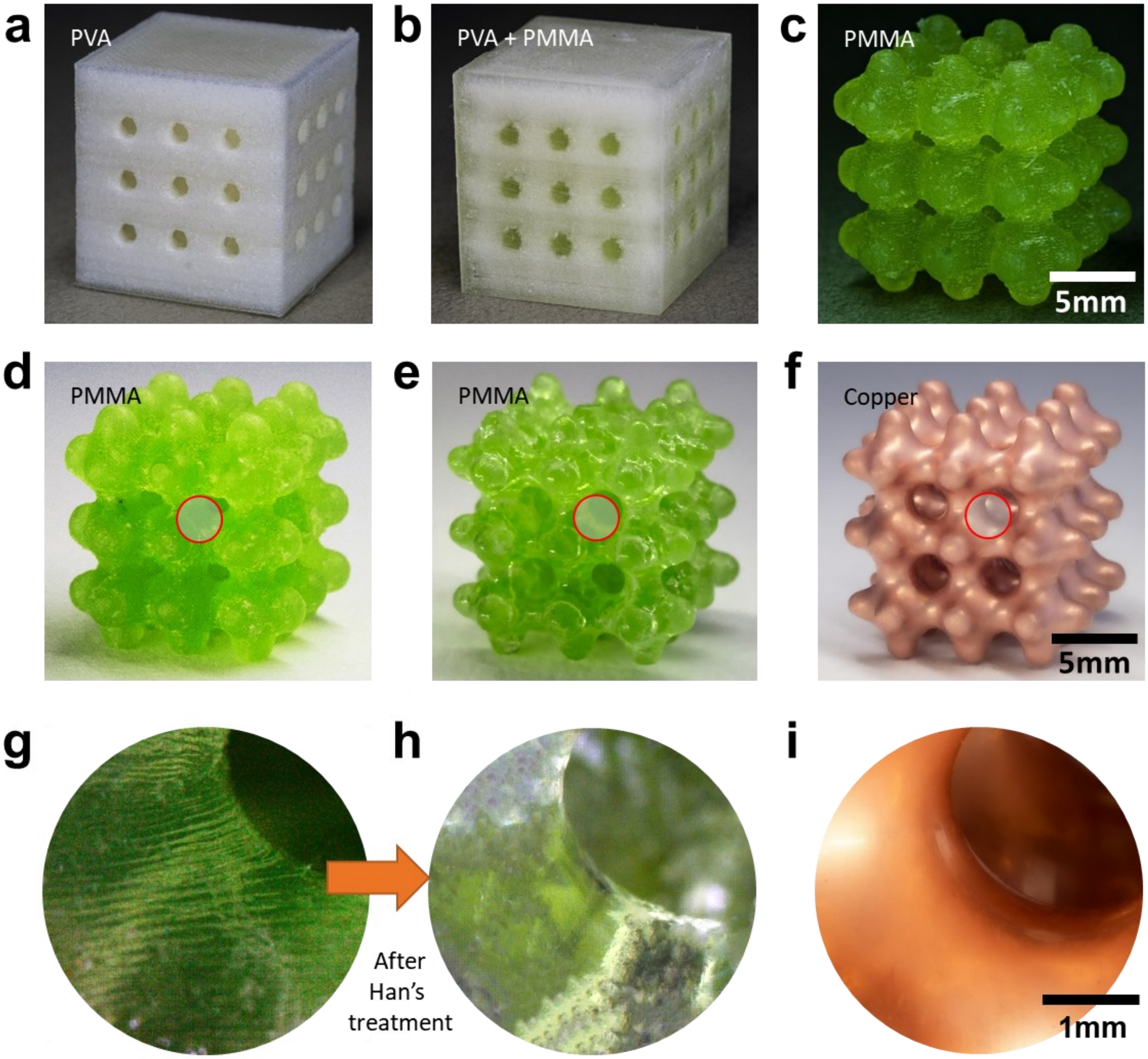
a
PVA
b
PVA + PMMA
c
PMMA
5mm
d
PMMA
e
PMMA
f
Copper
5mm
g
h
After
Han's
treatment
i
1mm

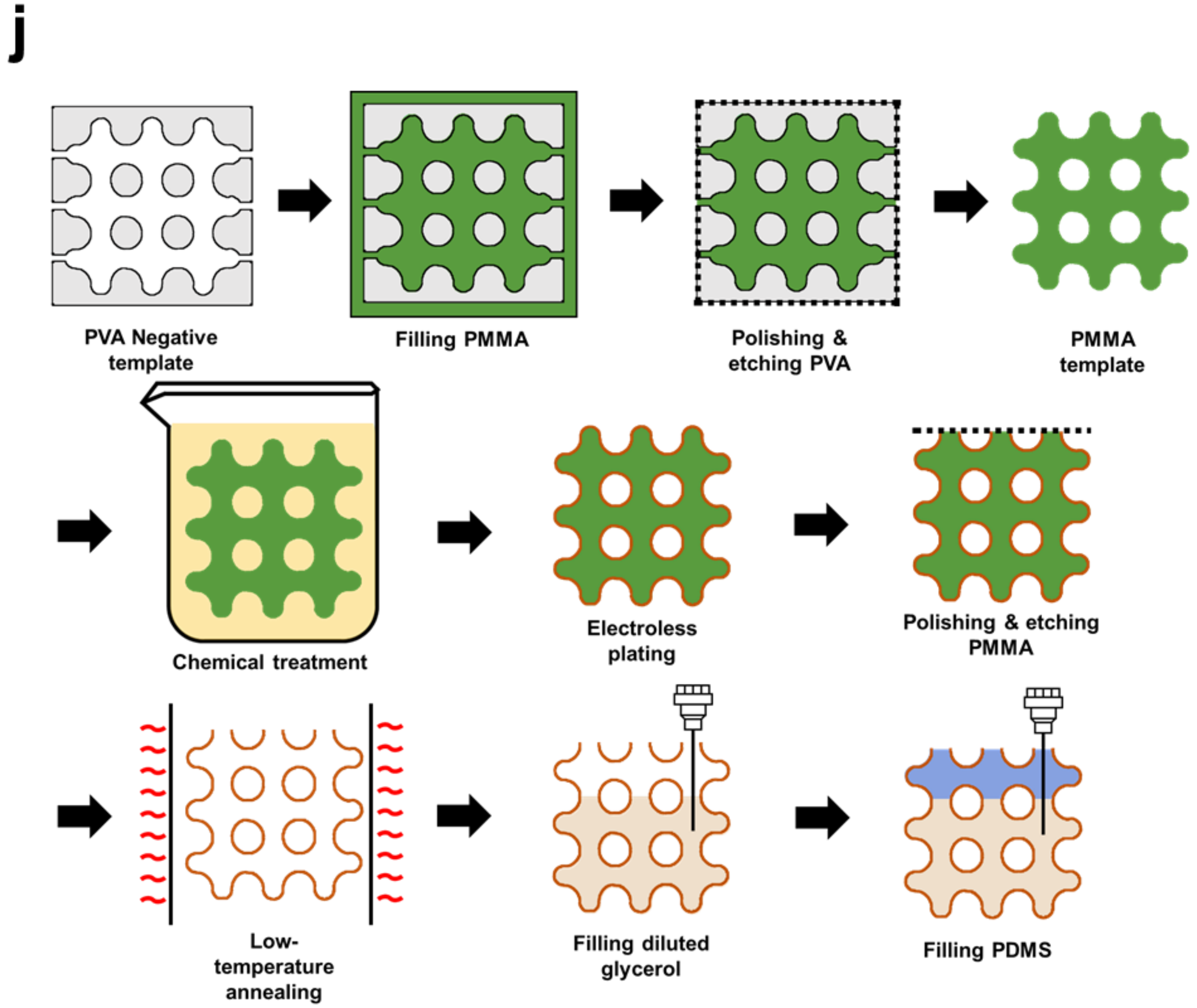


**Fig. S6. Fabrication of a shellular specimen for internal pressure tests.**

Images of **a**, a negative template of PVA as printed, **b**, the template with outer side faces polished to expose the interior PMMA, and **c**, the positive PMMA template obtained after PVA was etched out. **d**, A PMMA positive template with side caps ground like domes, **e**, the template with the surface smoothened through Han's treatment, **f**, a copper shellular specimen. **g**, **h**, and **i**, Magnified images of (**d**), (**e**), and (**f**), respectively. **j**, Schematics of an entire process conducted to obtain a shellular specimen.

A shellular specimen was obtained by etching out the PMMA interior through small holes, exposed by polishing the top face. Etching was performed by dipping in THF for 2 days. Figure S7a shows a Cu shellular with a polished top face.

According to Nakahara and Okinaka[9], the chemical reaction during electroless Cu plating produces hydrogen, causing a small portion of hydrogen to be co-deposited as gas bubbles along the Cu layer's grain boundaries, which substantially reduces its ductility. The hydrogen can be removed by low-temperature annealing. The ductility of the Cu shell is essential for cold stretching, which is a critical procedure for achieving high pressure resistance of the shellular pressure vessel. Therefore, we annealed all shellular specimens at 260°C for 3 h in an electric oven with an argon atmosphere. After that, the specimens were dipped in Anta-100 anti-tarnish solution (Young-In Plachem Co. Ltd., Korea) at 50 °C for 1 min and dried in the air to form a protective coating on the surfaces. The etching, annealing, and anti-tarnish coating procedures were conducted quickly without any intermission to minimize the possibility of surface corrosion.

Since the shellular specimens had multiple holes on the top face, the holes should be sealed except for one connected with an external tubular needle for the internal pressure tests. In addition, the sealed areas should have higher resistance against internal pressure than the remaining area of the shell without any effect on the intrinsic pressure resistance of the shellular. Because the *P*-shellular specimens were very delicate in geometry, the sealing procedure was technically challenging. The sealing procedure was as follows.

For hermetical sealing of the shellular specimen, the inner space of the top layer with the holes was filled using polydimethylsiloxane (PDMS), while 60 % glycerol aqueous solution was used to fill the void underneath the PDMS sealing. At the concentration of the glycerol solution, its density was higher than that of the PDMS before hardening, thus allowing the raw unhardened PDMS to float above the glycerol solution, as shown in Fig. S7b. The solution was also used as the medium for transferring internal pressure because it is chemically inactive and easy to handle. For this purpose, each of the empty shellulars was placed within a small 3D-printed box to hold the specimen with a tubular needle attached as shown in the bottom left photo of Fig. S7d, and the

void inside the shellular was then filled with 1.1 ml of glycerol solution. The amount of solution was equal to 2/3 of the overall volume of the inner space of each shellular specimen, which was calculated from the numerical model for the FEA in "Finite Element Analyses" section.

PDMS sealing was performed using SYLGARD™ (Dow Chemical Co.). Sylgard 184-A was mixed with hardener Sylgard 184-B in a weight ratio of 10:1 before being injected to fill the void in the top layer above the glycerol aqueous solution inside each shellular. When hardened at room temperature, the needle used to inject the glycerol solution was kept being inserted through the PDMS layer to add the glycerol solution into a vacant space that was likely formed above the underlying glycerol solution because the PDMS shrank as it hardened. During the main experiment, the needle was attached to a syringe filled with glycerol solution and used as a channel to apply internal pressure. Figures S7c and d show schematics of the PDMS seal resisting the internal pressure applied to the inner surface of each shellular via the glycerol aqueous solution and the shellular specimens ready for use in the internal pressure tests, respectively. Since PDMS has an extremely low elastic modulus in comparison to that of the Cu shell, no stress concentration was observed at contact with the thin shells. This was verified using finite element analysis in advance ("Effects of PDMS Seals" section).

Since the shell of a double-chambered model is not free of self-intersection, it is no longer possible to prepare the specimens through single plating and etching, as done for the single-chambered specimens. Thus, a double-chambered specimen was prepared by plugging the openings on the outer faces of a single-chambered specimen using polystyrene beads (2 mm diameter) coated with PDMS after making holes on the interior shell for simultaneous pressurization of both sub-volumes during the internal pressure tests. The holes were made by piercing the interior interfacial shell using the needle.

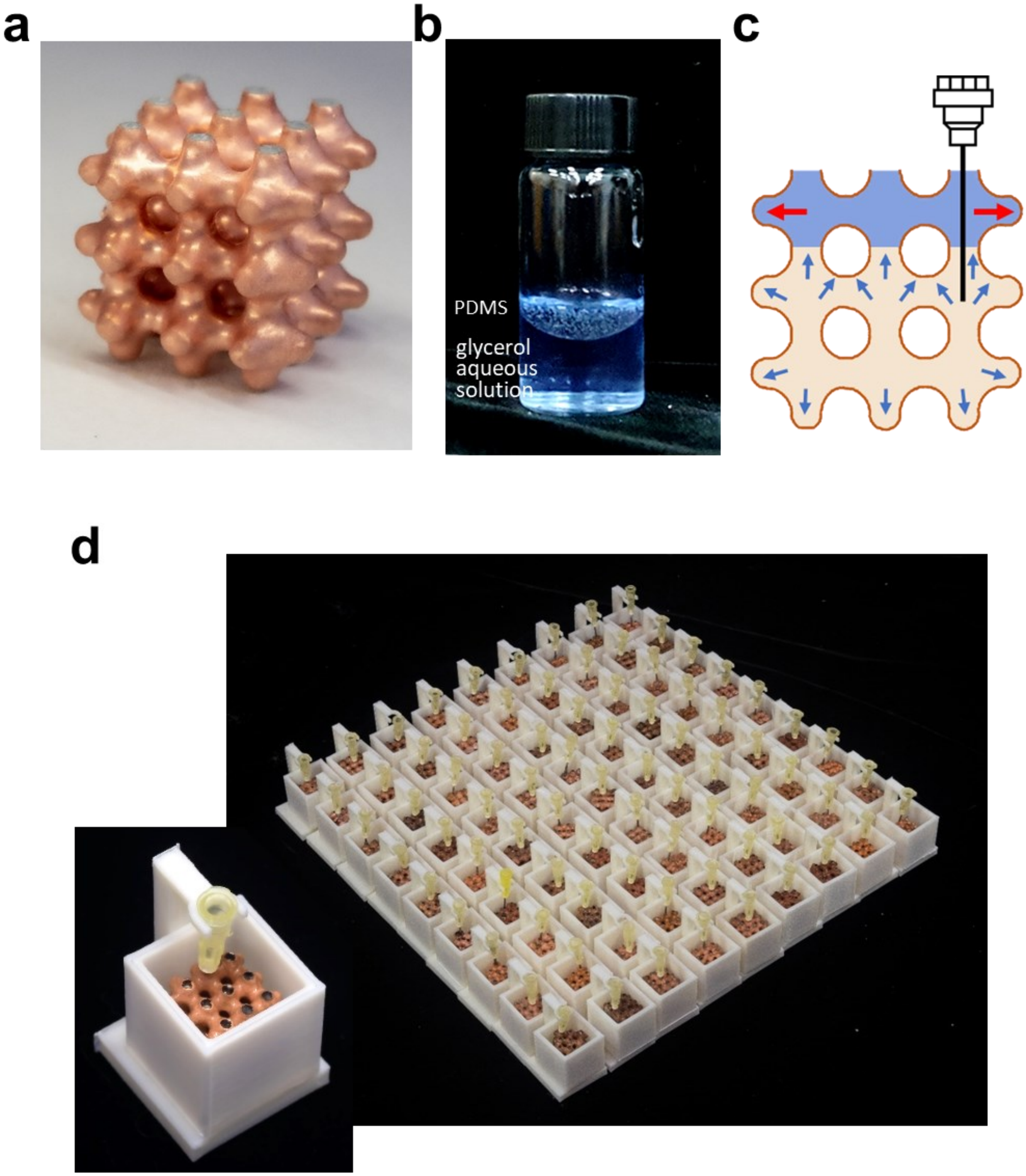


**Fig. S7. Hermitical PDMS sealing for internal pressure tests.**

Images of **a**, a copper shellular with the top face polished, **b**, raw (unhardened) PDMS floating on glycerol aqueous solution. **c**, Schematics of the PDMS seal resisting the internal pressure applied to each shellular via the glycerol aqueous solution and **d**, shellular specimens ready for the internal pressure test.

**Supplementary Note 7:**

**Measurement of Yield Pressures of Shellulars**

The internal cavity of each specimen was filled with glycerol aqueous solution, and the specimen was attached to a syringe filled with the identical solution. The syringe attached to the specimen was then mounted on top of a rectangular frame, as shown in Figs. 3a and b, for the internal pressure test. The frame was mounted on an electro-hydraulic material test system, INSTRON 8872. Figure S8 depicts the test system used to measure the yield pressures of the shellular specimens. To apply such high pressures that the specimens with thick shells such as $t/D \approx 0.01$ yield, a stainless steel syringe (VCDS10, VMATIC Co., China) with a high-pressure male Luer Lock connector 80353 (Qosina Corp., NY, USA) adhesively bonded to a nozzle using Loctite® Epoxy Instant Mix™ (Henkel Corp., USA) was used. A needle (20 G, PrecisionGlide™, Becton-Dickinson & Co., USA) whose end was inserted into a specimen through PDMS sealing was attached to the Luer Lock connector.

The pressure was applied by pushing the syringe's plunger at a constant displacement rate of 0.01 mm/s. The plunger's displacement was measured using a linear variable displacement transducer (LVDT) built into the test system. In addition to the built-in load cell, a small-capacity load cell (CWFS-20, Bongshin Loadcell Co., Ltd., Republic of Korea) with a load capacity of 200 N was placed under the frame to measure the applied load more precisely. In cases of the specimens with $t/D$ values higher than 0.00509, whose estimated loads exceeded 200 N, another small-capacity load cell (CWFS-100, Bongshin Loadcell Co., Ltd., Republic of Korea) with a load capacity of 1 kN was used for load measurement. The load and displacement data were acquired using a strain amplifier (2311 Signal Conditioning Amplifier, Vishay Measurements Corp., USA) and data acquisition board (DT322 Data Translation, Spectrum Instrumentation Corp., USA), and stored in a personal computer. The load data were divided by the cross-sectional area of the syringe's plunger, the diameter of which was 14.8 mm, to convert them to the internal pressure applied to each shellular vessel specimen through the needle.

The initial yield point of the specimen was monitored in the load-displacement curve. To implement cold stretching after the initial yield, the pressure was further applied to induce overall plastic deformation in the shell until the displacement was at least doubled. Subsequently, to measure the yield pressure and failure pressure of the cold-stretched specimen, the pressure was fully released before being applied again until the specimen failed. Table S4 lists the dimensions and measured yield pressures of the shellular specimens.

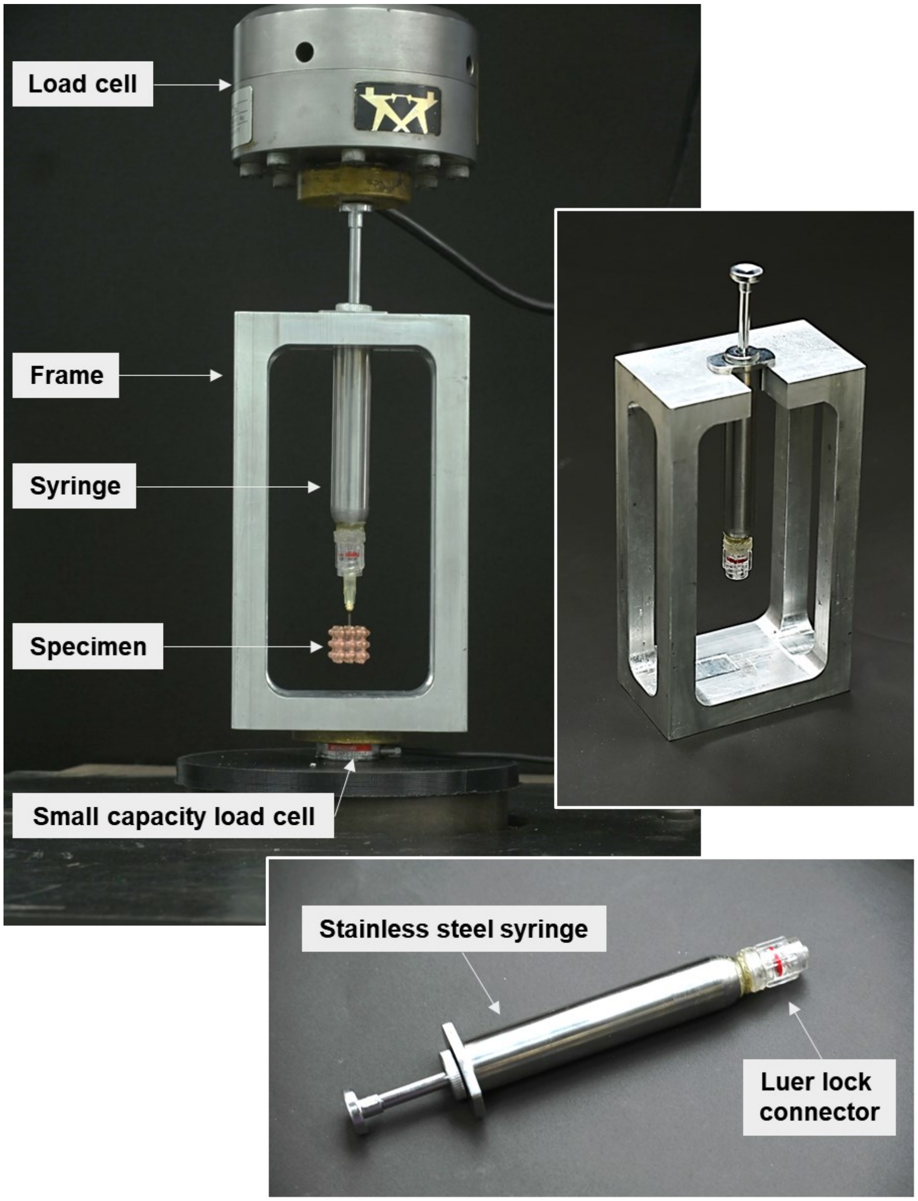


**Fig. S8. Test setup for measuring yield pressures of shellular pressure vessel specimens.**

**Supplementary Note 8:**

**Tensile Tests of Cu Foils**

After all the fabrication processes and conditions of shellular specimens, particularly plating and post-treatments for achieving uniform thickness and sufficient ductility, were set, the tensile properties of the Cu foils as the constituent material of the shellular specimens were measured. When we tried to perform Cu plating on a PMMA flat coupon, unlike the TPMS-shaped templates used for the fabrication of the shellulars, the plated Cu layer detached early from the surface before reaching the desired thickness, due to a coplanar stress mismatch occurring between the Cu layer and the underlying PMMA caused by the high plating temperature of 70 °C. Thus, we prepared a PMMA bar with a circular cross-section of 6.37 mm diameter (to obtain 20 mm long coupons). Surface treatment and electroless plating were conducted in the same way as that performed for the shellular specimens. Before etching out the PMMA template, one cut was made along the longitudinal direction on the plated bar using a scalpel, while several cuts were made around the circumference at a constant distance of 3 mm. Subsequently, similar to the fabrication of the shellular specimens, the coupons were annealed at 260°C for 3 h in an electric oven with an argon atmosphere as they were pressed between two glass slides to obtain flat coupons of Cu foil in a size of 3 mm × 20 mm. The other procedures were also the same as those conducted for the shellular specimens. Tensile tests were conducted for the Cu foil coupons with various thicknesses. See Han et al.[6] for the technical details of the test system and procedure. The only difference was in the strain measurement method. Instead of using the overall displacement estimated from the step motor control signal, a series of photographs were taken for the gauge section during each tensile test. The number of pixels between the two joints with the stainless-steel frame in the digital images was counted to precisely measure the strain.

Figures. S9a and b show a specimen installed for the tensile test and the measured stress-strain curves of the Cu foils with five different thicknesses (5, 16.25, 27.5, 38.75, 50 μm), respectively. Figures. S9c and d show that the elongations and yield strength are in the ranges of 2.5 ± 1.5 % and 120 ± 20 MPa regardless of the Cu foil's thickness, respectively.

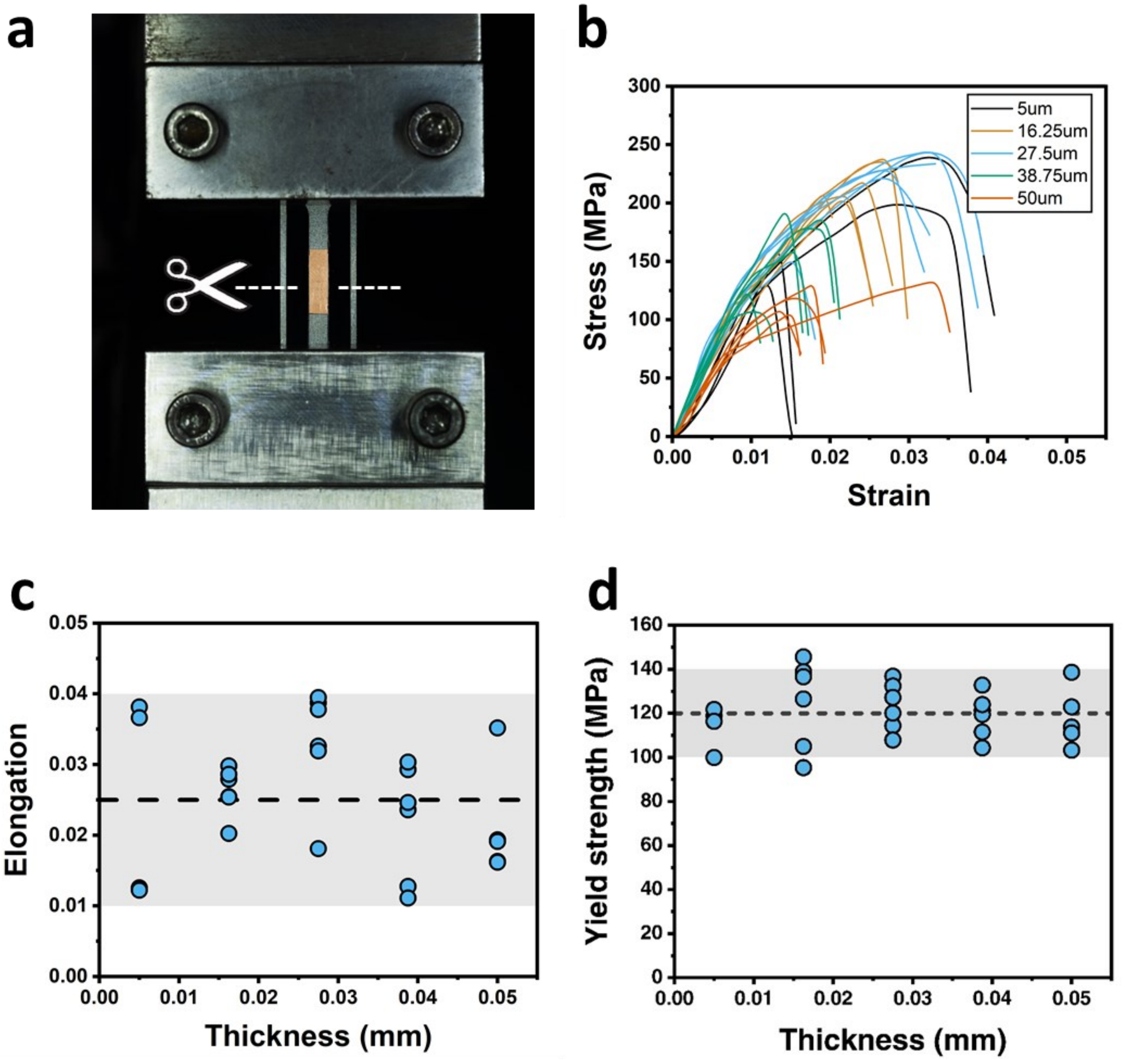


**Fig. S9. Mechanical properties measured from tensile tests for copper foils.**

**a**, An installed copper foil specimen for the tensile test, **b**, measured stress-strain curves. **c**, Elongations and **d**, yield strengths plotted against the foil thickness.

**Supplementary Note 9:**

**Surface Roughness of Cu Shells**

The Cu coupons prepared for the tensile tests were cut into squares of 5 mm × 5 mm and used to measure the surface profile using a white light scanning interferometer (NV-E1000, NanoSystem Co., Ltd, South Korea). Figure S10 shows the results measured from a 4.06 μm thick Cu coupon. Specifically, Figures S10a and b show the profiles in the surface area of 621 μm × 466 μm and along the x-axis across the middle line, respectively, measured from the inner surface from which the plating reaction was initiated on the PMMA template. Figures. S10c and d show the profiles in another surface area, measured from an outer surface at which the plating reaction was terminated. Two surface profiles denoted as "Original" and "Detrended" are plotted in each of Figs. S10b and d. The latter means that the surface profile was treated by a function of MATLAB®, widely used to remove long-term trends for emphasizing short-term changes. That is, the detrended profile shows the intrinsic roughness without the effect of creases in the thin shell. The maximum peak-to-valley values as a scale of the roughness of the inner and outer surfaces were 3.22 μm and 1.89 μm, respectively. The inner surface was rougher than the outer surface.

Figures S10 e to h show the results measured from a 30.10 μm thick shell in the same manner as Figs. S10a to d. The maximum peak-to-valley values as a scale of the roughness of the inner and outer surfaces were 3.43 μm and 10.25 μm, respectively. The outer surface was much rougher than the inner surface. The results shown in Fig. S10 suggest that, first, the inner surface roughness was due to the surface pretreatment applied to promote electroless plating. Consequently, the roughness scaled by the maximum peak-to-valley value was almost constant at ~ 4 μm, regardless of the plating time and Cu foil thickness. Second, the outer surface roughness was initially lower than that of the inner surface; however, the roughness increased with plating time as nodules formed and grew in size. The phenomenon of nodule formation and its growth with plating time is well-known in electroless Cu plating[10].

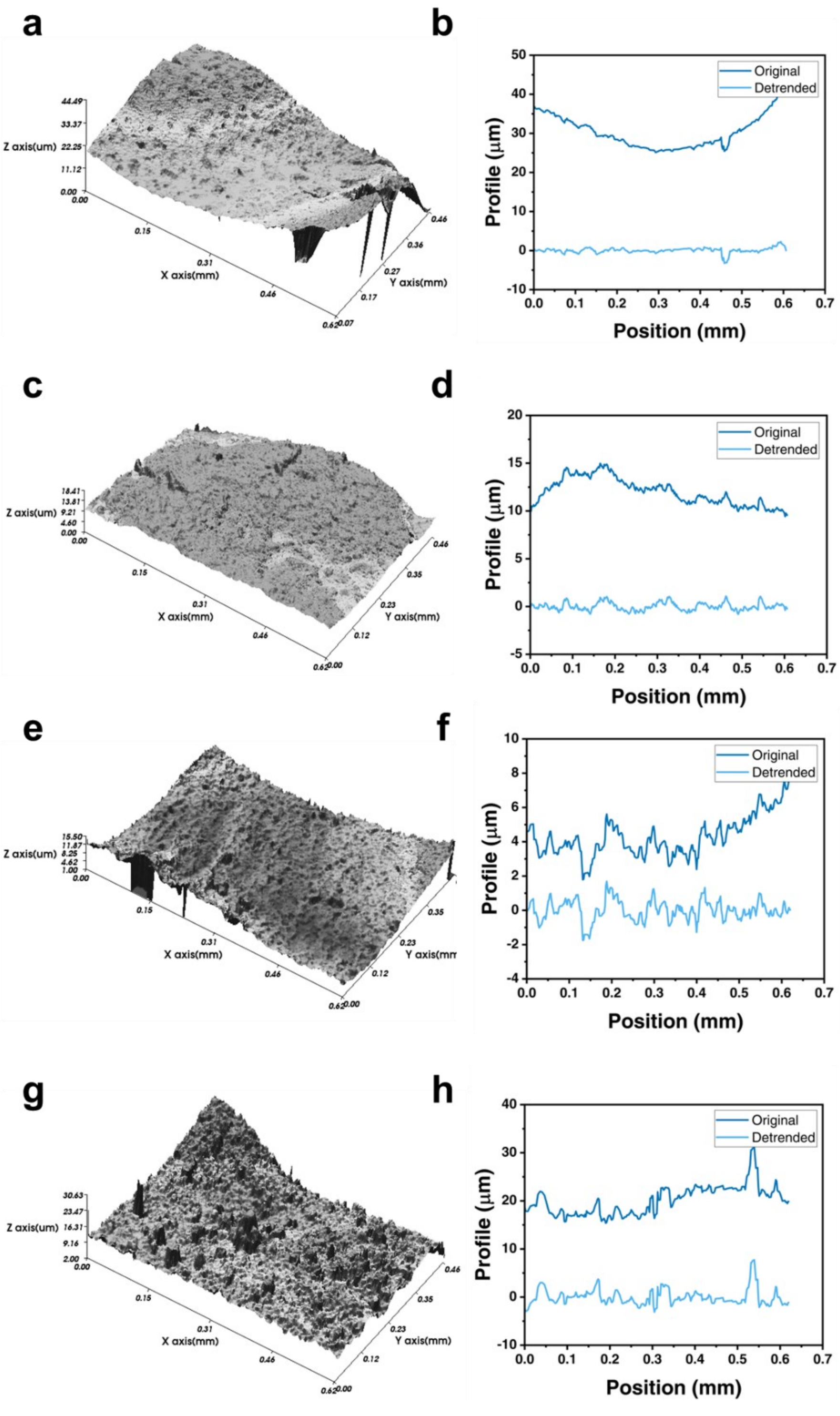


**Fig. S10. Surface profiles for a copper shell.**

Surface profiles and roughness measured from a copper shell with a thickness of 4.06μm **a**, **b, c, d** and with a thickness of 30.10μm **e, f, g, h.** in both surfaces.

**Supplementary Note 10:**

**Effects of PDMS Seals**

Finite element analysis was performed based on the shellular model that was used for the experimental internal pressure tests. The meshes and element type were the same as those shown in Fig. S2a in "Finite Element Analyses" section with the thinnest shell of $t = 0.001\ D$, but the overall shape was cold-stretched and the nine sealing caps on the top face were removed, and the internal cavity of the top one of three layers was filled with PDMS. The PDMS was assumed to be linear elastic with Young's modulus of $E = 13.2$ MPa, which was overestimated by ten times that of the value reported for the same PDMS (Sylgard 184) for conservative estimation[11]. Figures. S11a and b show the von Mises stresses distributed on the cold-stretched model with the PDMS seal under internal pressure of $P = 0.002\ \sigma_o$ and $0.0023\ \sigma_o$, respectively. Both pressures were higher than the pressure resistance measured from the real specimens with $t/D = 0.001$, shown in Fig. 3e in the main texts. However, no sign of stress concentration due to the PDMS seal was observed. Thus, it was verified that the PDMS seal had no effect on the yield pressure of the shellular vessel.

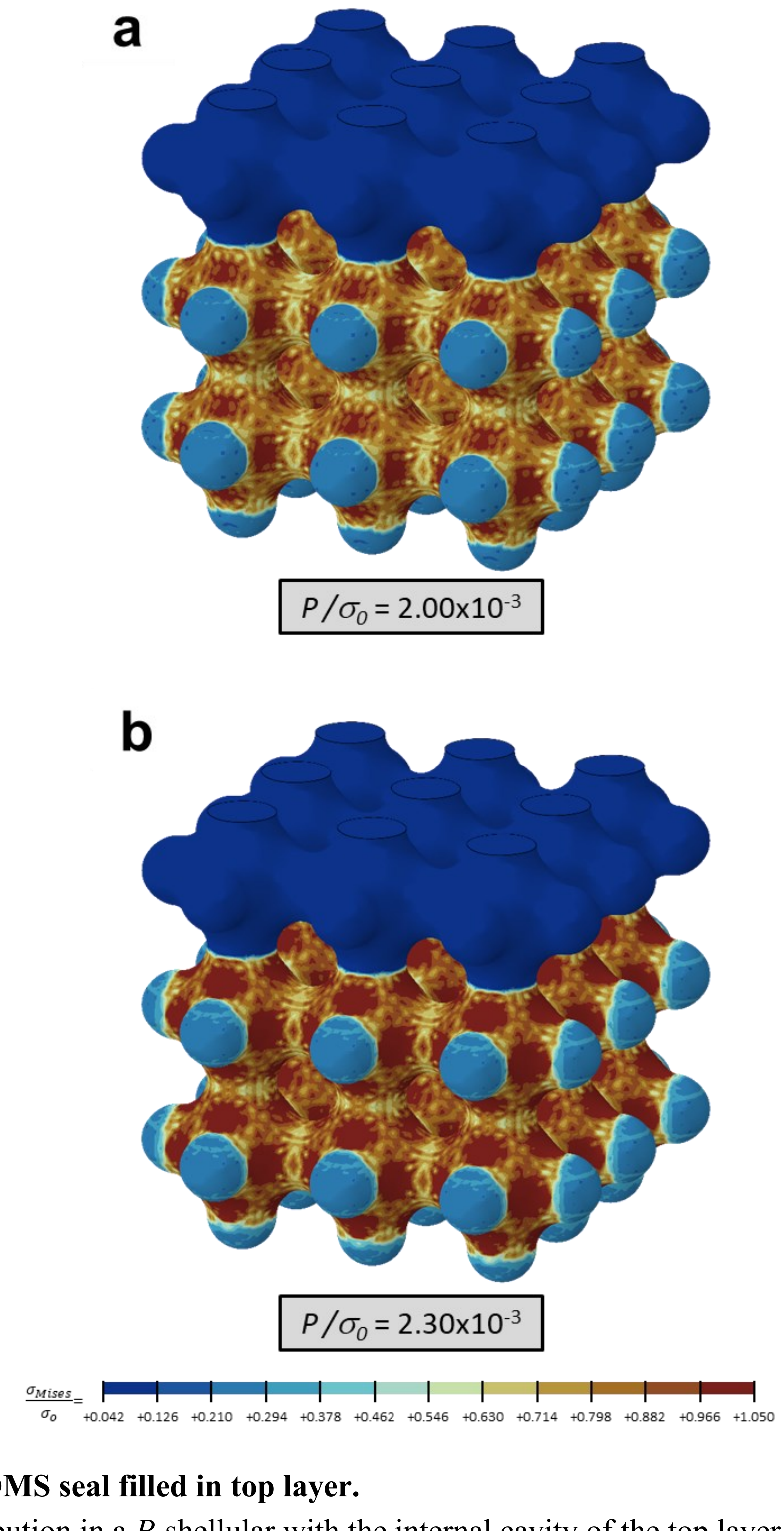


**Fig. S11. Effects of PDMS seal filled in top layer.**

Von Mises stress distribution in a *P*-shellular with the internal cavity of the top layer filled with PDMS, under internal pressures of **a**, $P = 0.002\ \sigma_o$ and **b**, $0.0023\ \sigma_o$

**Supplementary Reference**


1. Lee. M. G., Lee. J. W., Han. S. C., Kang. K, Mechanical analyses of “Shellular”, an ultralow-density material, Acta Materialia 103, 595-607 (2016)
2. Kolesnikova, T., Wu, C. H., Han, S. C. & Kang, K. Failure of P-surfaced shellular subjected to internal pressure. *AIP Adv.* **9**, 025010; 10.1063/1.5066578 (2019).
3. Brakke, K. A. The surface evolver. *Exp. Math*. **1:2**, 141-165 (1992).
4. Wu, C. H. *Failure Study of Shellulars under Internal Pressure*. Master thesis, Graduate School, Chonnam National University (2019).
5. Dassault Systèmes Simulia Corp (2006). Abaqus Analysis User′s Manual, Available at: https://classes.engineering.wustl.edu/2009/spring/mase5513/abaqus/docs/v6.6/books/usb/default.htm?startat=pt06ch23s06alm15.html (Accessed: 19th September 2023)
6. Dassault Systèmes Simulia Corp (2006). ABAQUS Theory Manual, Available at: https://classes.engineering.wustl.edu/2009/spring/mase5513/abaqus/docs/v6.6/books/stm/default.htm?startat=ch03s06ath79.html (Accessed: 19th September 2023)
7. Nguyen, B. D., Han, S. C., Jeong, Y. C. & Kang, K. Design of the P-surfaced shellular, an ultra-low density material with micro-architecture. *Comput. Mater. Sci.* **139**, 162–178 (2017).
8. Han, S.C. & Kang, K. Another stretching-dominated architecture, shellular. *Mater. Today.* **31**, 31-38 (2019).
9. Nakahara, S. & Okinaka, Y. Microstructure and mechanical properties of electroless copper deposites. *Annu. Rev. Mater. Sci.* **21**, 93-129 (1991).
10. Cui, X., Hutt, D. A. & Conway, P. P. An investigation of electroless copper films deposited on glass. *In Proceedings of 2nd IEEE Electronics System-Integration Technology Conference,* 105-110 (2008).

11. Johnston, I. D., McCluskey, D. K., Tan, C. K. L. & Tracey, M. C. Mechanical characterization of bulk Sylgard 184 for microfluidics and microengineering. *J. Micromech. Microeng.* **24**, 035017; 10.1088/0960-1317/24/3/035017 (2014).

**Table S4. Weights, dimensions, and yield pressures of shellular pressure vessel specimens used in internal pressure tests.**

| No. | Type | Status | Weight (g) | Thickness (mm) | Relative Thickness, *t/D* | Yield pressure (MPa) | Relative yield pressure, $P/\sigma_0$ |
|---|---|---|---|---|---|---|---|
| 1 | Single chamber | Original | 1.463E-01 | 8.086E-03 | 1.617E-03 | 1.708E-01 | 1.424E-03 |
| 2 | | | 2.773E-01 | 1.532E-02 | 3.064E-03 | 2.176E-01 | 1.813E-03 |
| 3 | | | 1.861E-01 | 1.028E-02 | 2.056E-03 | 3.373E-01 | 2.811E-03 |
| 4 | | | 1.535E-01 | 8.483E-03 | 1.697E-03 | 2.872E-01 | 2.393E-03 |
| 5 | | | 1.517E-01 | 8.381E-03 | 1.676E-03 | 2.606E-01 | 2.171E-03 |
| 6 | | | 2.364E-01 | 1.306E-02 | 2.613E-03 | 2.765E-01 | 2.304E-03 |
| 7 | | | 2.549E-01 | 1.409E-02 | 2.817E-03 | 1.883E-01 | 1.569E-03 |
| 8 | | | 2.645E-01 | 1.461E-02 | 2.923E-03 | 1.831E-01 | 1.526E-03 |
| 9 | | | 2.870E-01 | 1.586E-02 | 3.171E-03 | 4.805E-01 | 4.004E-03 |
| 10 | | | 7.327E-01 | 4.048E-02 | 8.097E-03 | 1.427E+00 | 1.189E-02 |
| 11 | | | 2.645E-01 | 1.461E-02 | 2.923E-03 | 5.145E-01 | 4.288E-03 |
| 12 | | | 7.935E-01 | 4.384E-02 | 8.768E-03 | 1.609E+00 | 1.341E-02 |
| 13 | | | 7.422E-02 | 4.100E-03 | 8.201E-04 | 1.361E-01 | 1.135E-03 |
| 14 | | | 7.276E-02 | 4.020E-03 | 8.040E-04 | 1.183E-01 | 9.862E-04 |
| 15 | | | 7.149E-02 | 3.950E-03 | 7.900E-04 | 1.438E-01 | 1.198E-03 |
| 16 | | | 5.653E-01 | 3.123E-02 | 6.246E-03 | 4.757E-01 | 3.964E-03 |
| 17 | | | 5.282E-01 | 2.918E-02 | 5.836E-03 | 4.171E-01 | 3.476E-03 |
| 18 | | | 8.415E-01 | 4.649E-02 | 9.298E-03 | 5.383E-01 | 4.486E-03 |
| 19 | | | 4.721E-01 | 2.608E-02 | 5.216E-03 | 5.381E-01 | 4.484E-03 |
| 20 | | | 4.550E-01 | 2.514E-02 | 5.028E-03 | 7.927E-01 | 6.606E-03 |
| 21 | | | 4.113E-01 | 2.273E-02 | 4.545E-03 | 4.486E-01 | 3.739E-03 |
| 22 | | | 8.987E-01 | 4.966E-02 | 9.931E-03 | 1.759E+00 | 1.466E-02 |
| 23 | | | 4.700E-01 | 2.597E-02 | 5.194E-03 | 4.852E-01 | 4.043E-03 |
| 24 | | | 4.680E-01 | 2.586E-02 | 5.172E-03 | 6.165E-01 | 5.137E-03 |
| 25 | | | 4.620E-01 | 2.553E-02 | 5.106E-03 | 5.049E-01 | 4.208E-03 |
| 26 | | | 5.693E-01 | 3.145E-02 | 6.291E-03 | 6.676E-01 | 5.563E-03 |
| 27 | | | 5.452E-01 | 3.013E-02 | 6.025E-03 | 2.445E-01 | 2.038E-03 |

| | | | | | | | |
|---|---|---|---|---|---|---|---|
| 28 | | | 2.903E-01 | 1.604E-02 | 3.208E-03 | 2.578E-01 | 2.148E-03 |
| 29 | | | 5.773E-01 | 3.190E-02 | 6.379E-03 | 2.569E-01 | 2.141E-03 |
| 30 | | | 5.582E-01 | 3.084E-02 | 6.169E-03 | 2.218E-01 | 1.848E-03 |
| 31 | | | 6.636E-01 | 3.666E-02 | 7.333E-03 | 2.081E-01 | 1.734E-03 |
| 32 | | | 5.313E-01 | 2.935E-02 | 5.871E-03 | 2.438E-01 | 2.032E-03 |
| 33 | | | 3.862E-01 | 2.134E-02 | 4.268E-03 | 2.463E-01 | 2.052E-03 |
| 34 | | | 5.833E-01 | 3.223E-02 | 6.445E-03 | 1.140E+00 | 9.499E-03 |
| 35 | | | 3.816E-01 | 2.108E-02 | 4.217E-03 | 2.213E-01 | 1.845E-03 |
| 36 | | | 3.831E-01 | 2.117E-02 | 4.234E-03 | 2.870E-01 | 2.392E-03 |
| 37 | | | 3.796E-01 | 2.097E-02 | 4.194E-03 | 2.418E-01 | 2.015E-03 |
| 38 | | | 2.870E-01 | 1.586E-02 | 3.171E-03 | 1.941E-01 | 1.617E-03 |
| 39 | | | 4.989E-01 | 2.756E-02 | 5.513E-03 | 1.838E-01 | 1.532E-03 |
| 40 | | | 6.283E-01 | 3.471E-02 | 6.943E-03 | 1.969E-01 | 1.641E-03 |
| 41 | | | 5.308E-01 | 2.933E-02 | 5.866E-03 | 2.082E-01 | 1.735E-03 |
| 42 | | | 4.944E-01 | 2.731E-02 | 5.463E-03 | 6.241E-01 | 5.200E-03 |
| 43 | | | 5.070E-01 | 2.801E-02 | 5.602E-03 | 1.243E-01 | 1.036E-03 |
| 44 | | | 4.672E-01 | 2.582E-02 | 5.163E-03 | 5.528E-01 | 4.606E-03 |
| 45 | | | 5.735E-01 | 3.169E-02 | 6.337E-03 | 9.995E-01 | 8.329E-03 |
| 46 | | | 1.294E+00 | 7.148E-02 | 1.430E-02 | 1.440E+00 | 1.200E-02 |
| 47 | | | 1.243E+00 | 6.868E-02 | 1.374E-02 | 1.711E+00 | 1.426E-02 |
| 48 | | | 1.198E+00 | 6.617E-02 | 1.323E-02 | 1.019E+00 | 8.492E-03 |
| 49 | | | 2.000E+00 | 1.105E-01 | 2.210E-02 | 2.990E+00 | 2.491E-02 |
| 50 | | | 2.080E-01 | 1.149E-02 | 2.299E-03 | 3.978E-01 | 3.315E-03 |
| 51 | | | 1.991E+00 | 1.100E-01 | 2.200E-02 | 9.478E-01 | 7.898E-03 |
| 52 | | | 2.000E+00 | 1.105E-01 | 2.210E-02 | 2.412E+00 | 2.010E-02 |
| 53 | | | 1.897E+00 | 1.048E-01 | 2.096E-02 | 9.426E-01 | 7.855E-03 |
| 54 | | | 1.278E+00 | 7.063E-02 | 1.413E-02 | 1.235E+00 | 1.029E-02 |
| 55 | | | 1.067E-01 | 5.893E-03 | 1.179E-03 | 4.190E-02 | 3.492E-04 |
| 56 | | | 9.843E-02 | 5.439E-03 | 1.088E-03 | 6.791E-02 | 5.659E-04 |
| 57 | | | 1.847E-01 | 1.020E-02 | 2.040E-03 | 6.733E-02 | 5.610E-04 |
| 58 | | | 2.053E-01 | 1.134E-02 | 2.268E-03 | 1.559E-01 | 1.300E-03 |
| 59 | | | 9.583E-02 | 5.295E-03 | 1.059E-03 | 7.758E-02 | 6.465E-04 |

| | | | | | | | |
|---|---|---|---|---|---|---|---|
| 4-CS | | Cold-stretched | 1.535E-01 | 8.483E-03 | 1.697E-03 | 3.460E-01 | 2.884E-03 |
| 9-CS | | | 2.870E-01 | 1.586E-02 | 3.171E-03 | 6.221E-01 | 5.184E-03 |
| 10-CS | | | 7.327E-01 | 4.048E-02 | 8.097E-03 | 1.662E+00 | 1.385E-02 |
| 11-CS | | | 2.645E-01 | 1.461E-02 | 2.923E-03 | 7.200E-01 | 6.000E-03 |
| 12-CS | | | 7.935E-01 | 4.384E-02 | 8.768E-03 | 1.971E+00 | 1.642E-02 |
| 15-CS | | | 7.149E-02 | 3.950E-03 | 7.900E-04 | 1.558E-01 | 1.298E-03 |
| 22-CS | | | 8.522E-01 | 4.709E-02 | 9.417E-03 | 2.201E+00 | 1.834E-02 |
| 34-CS | | | 5.833E-01 | 3.223E-02 | 6.445E-03 | 1.285E+00 | 1.071E-02 |
| 42-CS | | | 4.944E-01 | 2.731E-02 | 5.463E-03 | 1.055E+00 | 8.791E-03 |
| 45-CS | | | 5.735E-01 | 3.169E-02 | 6.337E-03 | 1.361E+00 | 1.134E-02 |
| 47-CS | | | 1.243E+00 | 6.868E-02 | 1.374E-02 | 2.526E+00 | 2.105E-02 |
| 50-CS | | | 2.080E-01 | 1.149E-02 | 2.299E-03 | 5.064E-01 | 4.220E-03 |
| 52-CS | | | 2.000E+00 | 1.105E-01 | 2.210E-02 | 3.654E+00 | 3.045E-02 |
| 60 | Double chamber | Original | 5.633E-01 | 3.112E-02 | 6.224E-03 | 1.529E-01 | 1.275E-03 |
| 61 | | | 2.645E-01 | 1.461E-02 | 2.923E-03 | 1.839E-01 | 1.533E-03 |
| 62 | | | 2.549E-01 | 1.409E-02 | 2.817E-03 | 1.524E-01 | 1.270E-03 |
| 63 | | | 2.856E-01 | 1.578E-02 | 3.156E-03 | 1.556E-01 | 1.297E-03 |
| 64 | | | 1.517E-01 | 8.381E-03 | 1.676E-03 | 1.448E-01 | 1.207E-03 |
| 65 | | | 1.463E-01 | 8.086E-03 | 1.617E-03 | 1.543E-01 | 1.286E-03 |
| 66 | | | 2.932E-01 | 1.620E-02 | 3.240E-03 | 2.601E-01 | 2.168E-03 |
| 67 | | | 1.580E-01 | 8.729E-03 | 1.746E-03 | 2.982E-01 | 2.485E-03 |
| 68 | | | 1.517E-01 | 8.381E-03 | 1.676E-03 | 2.183E-01 | 1.819E-03 |
| 69 | | | 1.535E-01 | 8.483E-03 | 1.697E-03 | 2.092E-01 | 1.743E-03 |
| 70 | | | 5.111E-01 | 2.824E-02 | 5.648E-03 | 2.006E-01 | 1.672E-03 |
| 71 | | | 7.255E-01 | 4.008E-02 | 8.017E-03 | 2.253E-01 | 1.878E-03 |
| 72 | | | 2.326E-01 | 1.285E-02 | 2.570E-03 | 3.810E-01 | 3.175E-03 |
| 73 | | | 1.026E-01 | 5.671E-03 | 1.134E-03 | 1.842E-01 | 1.535E-03 |
| 74 | | | 7.621E-01 | 4.211E-02 | 8.421E-03 | 8.632E-01 | 7.193E-03 |
| 75 | | | 3.511E-01 | 1.940E-02 | 3.880E-03 | 4.585E-01 | 3.821E-03 |
| 76 | | | 3.154E-01 | 1.742E-02 | 3.485E-03 | 2.183E-01 | 1.819E-03 |
| 77 | | | 5.050E-01 | 2.790E-02 | 5.580E-03 | 8.038E-01 | 6.699E-03 |
| 78 | | | 9.684E-01 | 5.351E-02 | 1.070E-02 | 1.620E+00 | 1.350E-02 |

| | | | | | | | |
|---|---|---|---|---|---|---|---|
| 79 | | | 5.886E-01 | 3.252E-02 | 6.504E-03 | 1.087E-01 | 9.061E-04 |
| 80 | | | 1.877E-01 | 1.037E-02 | 2.074E-03 | 6.272E-02 | 5.227E-04 |
| 81 | | | 1.667E-01 | 9.212E-03 | 1.842E-03 | 6.741E-02 | 5.617E-04 |
| 82 | | | 1.683E-01 | 9.297E-03 | 1.859E-03 | 2.572E-01 | 2.144E-03 |
| 83 | | | 1.363E-01 | 7.531E-03 | 1.506E-03 | 2.774E-01 | 2.312E-03 |
| 67-CS | | Cold-stretched | 1.580E-01 | 8.729E-03 | 1.746E-03 | 3.692E-01 | 3.077E-03 |
| 72-CS | | | 2.326E-01 | 1.285E-02 | 2.570E-03 | 5.314E-01 | 4.428E-03 |
| 73-CS | | | 1.026E-01 | 5.671E-03 | 1.134E-03 | 2.803E-01 | 2.335E-03 |
| 74-CS | | | 7.621E-01 | 4.211E-02 | 8.421E-03 | 1.396E+00 | 1.163E-02 |
| 75-CS | | | 3.511E-01 | 1.940E-02 | 3.880E-03 | 7.483E-01 | 6.236E-03 |
| 77-CS | | | 5.050E-01 | 2.790E-02 | 5.580E-03 | 1.154E+00 | 9.616E-03 |
| 78-CS | | | 9.684E-01 | 5.351E-02 | 1.070E-02 | 2.376E+00 | 1.980E-02 |
| 83-CS | | | 1.363E-01 | 7.531E-03 | 1.506E-03 | 3.089E-01 | 2.574E-03 |
