# Supplementary material for: A micro-architectured material as a pressure vessel for green mobility
Source: Nat Commun. 2024 Jan 8;15:353. doi: 10.1038/s41467-024-44695-4 (PMC10774278; doi:10.1038/s41467-024-44695-4)
Supplement: Supplementary file 3 — Description of Additional Supplementary Files [file 41467_2024_44695_MOESM3_ESM.pdf]

Description of Additional Supplementary Files

File Name: Supplementary Data 1
Description: Raw data and load-displacement curves measured from the internal pressure tests for all specimens.
